# Supplementary material for: Genome-wide association analysis of self-reported daytime sleepiness identifies 42 loci that suggest biological subtypes
Source: Nat Commun. 2019 Aug 13;10:3503. doi: 10.1038/s41467-019-11456-7 (PMC6692391; doi:10.1038/s41467-019-11456-7)
Supplement: Supplementary file 1 — Supplementary Information [file 41467_2019_11456_MOESM1_ESM.pdf]

**Supplementary Information**

# Genome-wide association analysis of self-reported daytime sleepiness identifies 42 loci that suggest biological subtypes

**Wang H et al.**

**Supplementary Figure 1.** Correlations between self-reported daytime sleepiness and other self-reported sleep traits in the UK Biobank.

**A.** Pairwise Spearman Phenotypic correlations among sleep traits.

| | Sleepiness | Insomnia | Sleep duration | Short sleep | Long sleep | Chronotype |
|---|---|---|---|---|---|---|
| Sleepiness | | 0.046 | -0.028 | 0.075 | 0.065 | 0.011 |
| Insomnia | *P* = 0* | | -0.192 | 0.265 | -0.006 | 0.000 |
| Sleep duration | $P = 1.0\times10^{-85}$ | *P* = 0* | | -0.981 | 0.582 | -0.022 |
| Short sleep | *P* = 0* | *P* = 0* | *P* = 0* | | NA | 0.033 |
| Long sleep | $P = 2.9\times10^{-317}$ | $P = 6.2\times10^{-4}$ | *P* = 0* | NA | | -0.031 |
| Chronotype | $P = 1.4\times10^{-10}$ | *P* = 0.95 | $P = 4.0\times10^{-60}$ | $P = 3.5\times10^{-63}$ | $P = 9.4\times10^{-66}$ | |

**B**. Pairwise genetic correlations calculated using LDSC among sleep traits.

| | Sleepiness | Insomnia | Sleep duration | Short sleep | Long sleep | Chronotype |
|---|---|---|---|---|---|---|
| Sleepiness | | 0.234 | -0.056 | 0.147 | 0.144 | 0.058 |
| Insomnia | $P = 9.8\times10^{-18}$ | | -0.509 | 0.644 | 0.007 | 0.030 |
| Sleep duration | *P* = 0.023 | $P = 2.5\times10^{-162}$ | | -0.893 | 0.694 | -0.068 |
| Short sleep | $P = 8.5\times10^{-7}$ | $P = 2.1\times10^{-230}$ | *P* = 0* | | -0.289 | 0.093 |
| Long sleep | $P = 1.6\times10^{-5}$ | 0.83 | $P = 1.6\times10^{-237}$ | $P = 1.1\times10^{-14}$ | | -0.023 |
| Chronotype | *P* = 0.002 | *P* = 0.16 | *P* = 0.005 | $P = 2.3\times10^{-4}$ | 0.44 | |

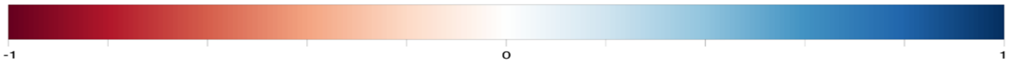


*P-values smaller than machine epsilon (the relative error due to rounding in computer floating point arithmetic) are shown as 0.

**Supplementary Figure 2.** QQ plot for primary genome-wide association analysis of self-reported daytime sleepiness adjusting for age, sex, genotyping array, top ten principal components and genetic relatedness matrix. GC: Genomic Control; LDSR: LD Score Regression.

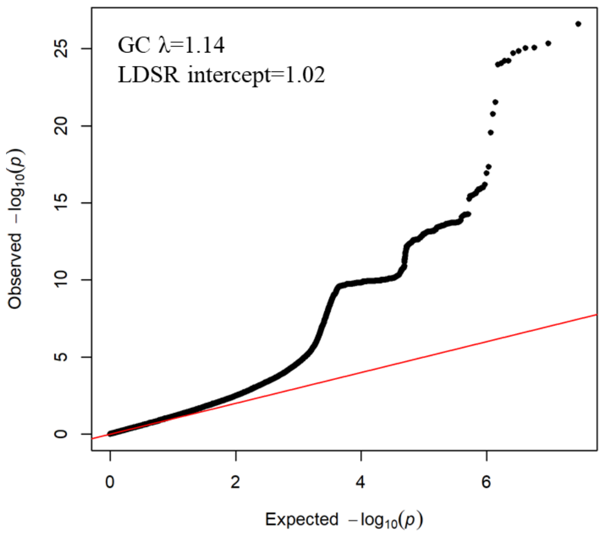

**Supplementary Figure 3.** Regional association plots of genome-wide significant loci ($5\times10^{-8}$) for self-reported daytime sleepiness adjusting for age, sex, genotyping array, top ten principal components and genetic relatedness matrix. Chromosomal position is indicated on the x-axis and –log10 p-values for each SNP (filled circles and squares) is indicated on the y-axis, with the lead SNP shown in purple (400kb window around lead SNP shown). Genes within the region are shown in the lower panel. The blue line indicates the recombination rate. Additional SNPs in the locus are colored according to linkage disequilibrium ($r^2$) with the lead SNP (estimated by LocusZoom based on the CEU HapMap haplotypes). Squares represent genotyped SNPs and circles represent imputed SNPs.

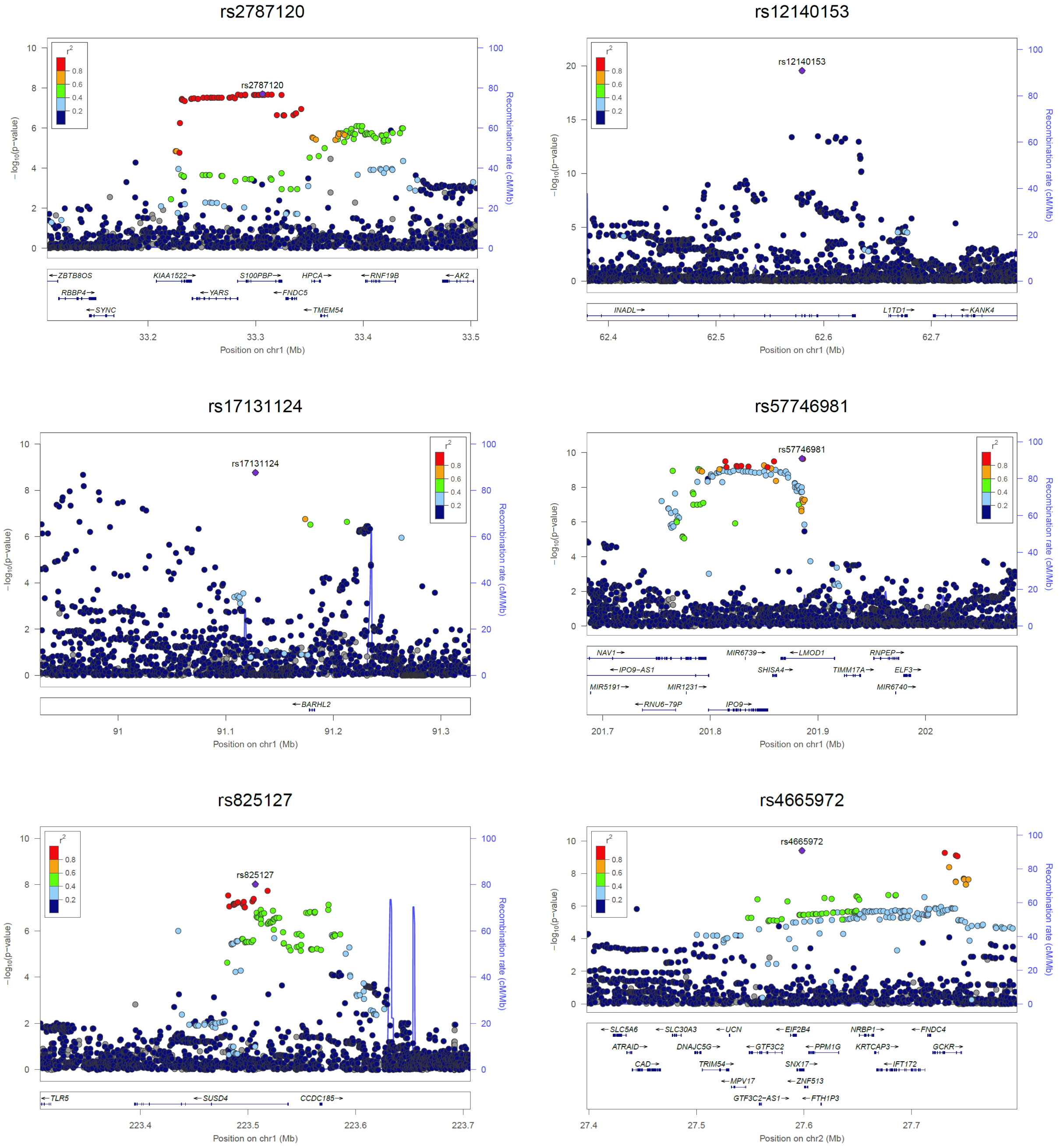

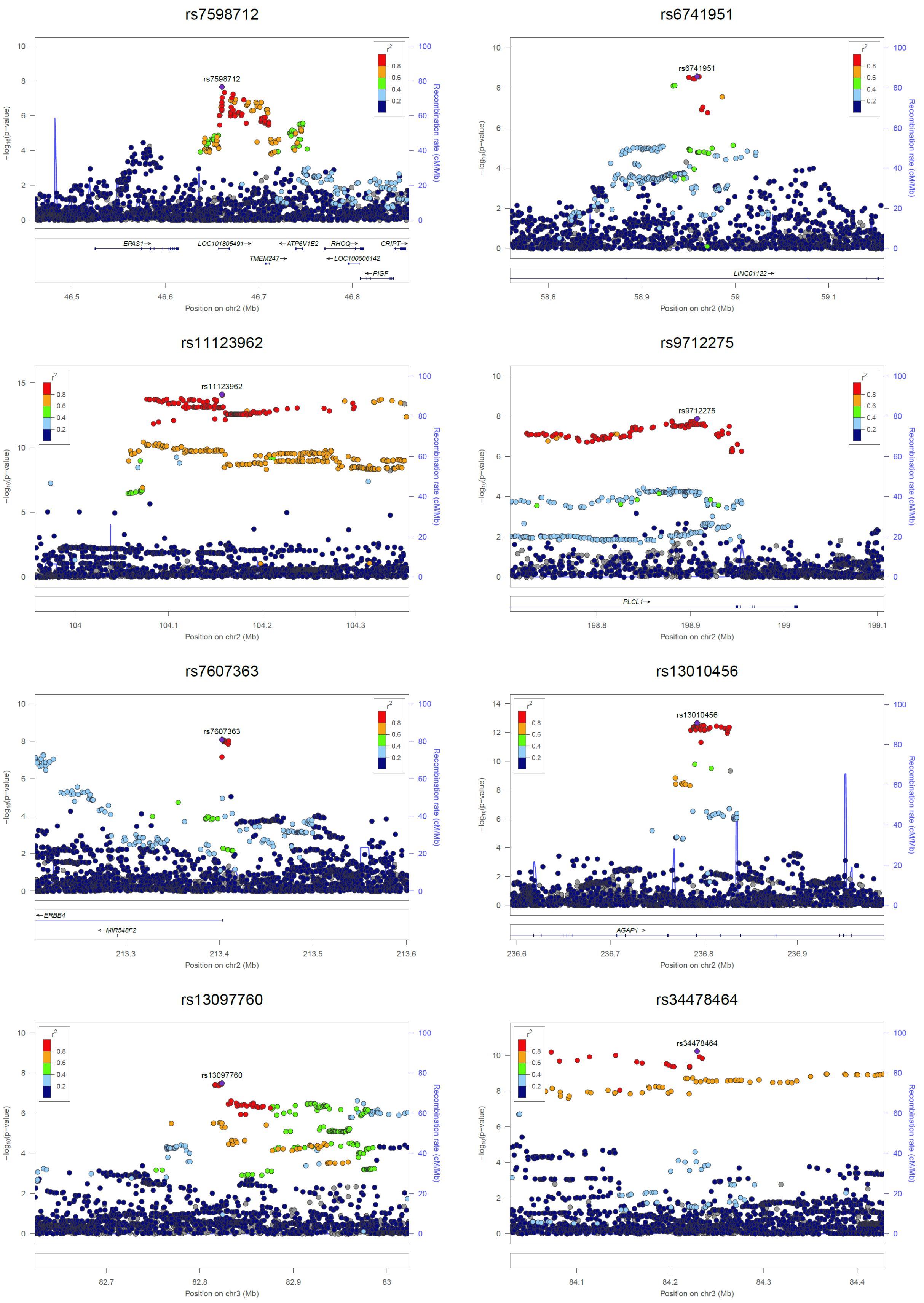
rs7598712
EPAS1→
LOC101805491→
←ATP6V1E2
RHOQ→
CRIPT→
TMEM247→
←LOC100506142
←PIGF
Position on chr2 (Mb)
rs6741951
LINC01122→
Position on chr2 (Mb)
rs11123962
Position on chr2 (Mb)
rs9712275
PLCL1→
Position on chr2 (Mb)
rs7607363
←ERBB4
←MIR548F2
Position on chr2 (Mb)
rs13010456
AGAP1→
Position on chr2 (Mb)
rs13097760
Position on chr3 (Mb)
rs34478464
Position on chr3 (Mb)
−log10(p−value)
Recombination rate (cM/Mb)
r2
0.8
0.6
0.4
0.2

rs960986

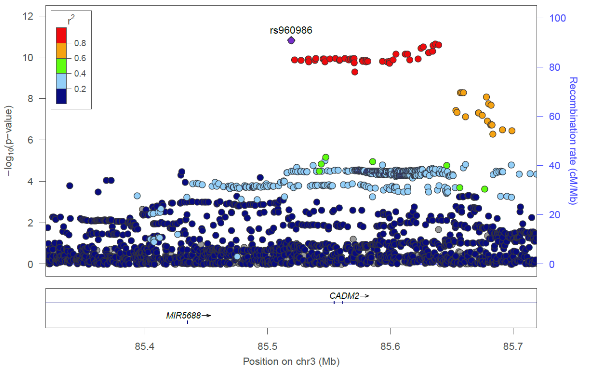


rs843372

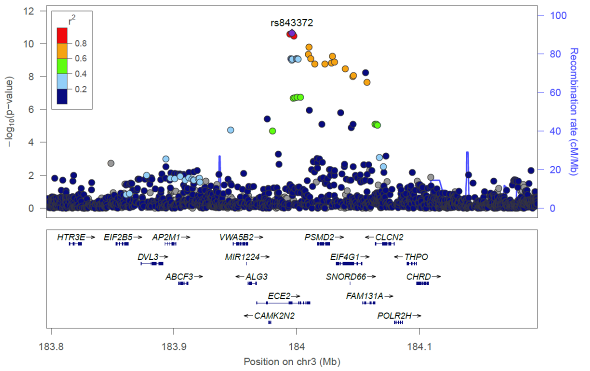


rs11942333

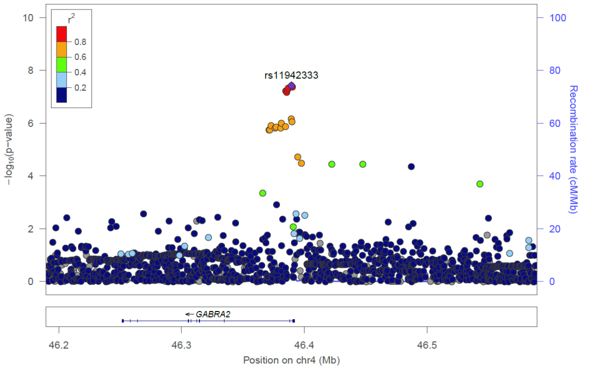


rs13135092

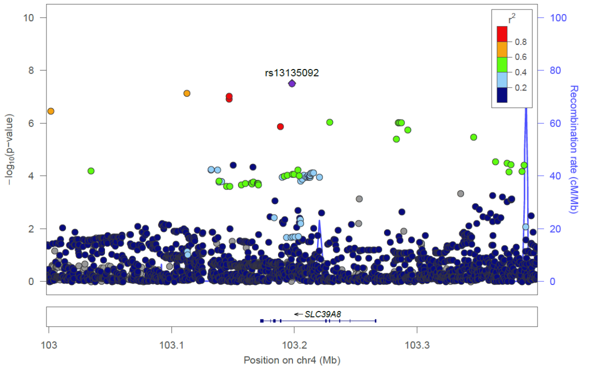


rs6897863

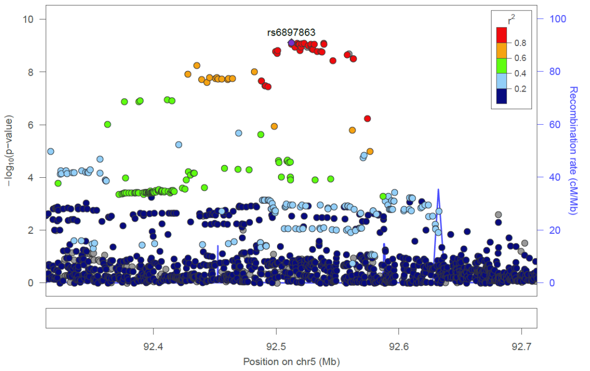


rs12153518

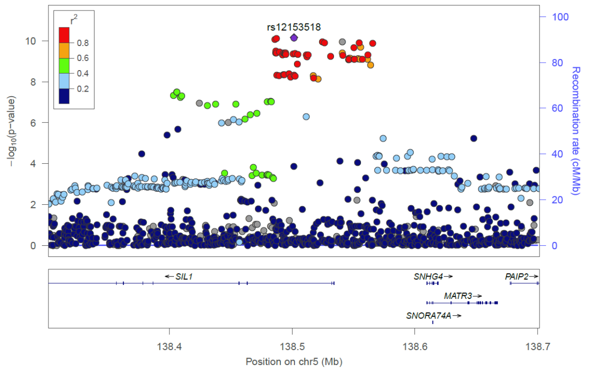


rs6923811

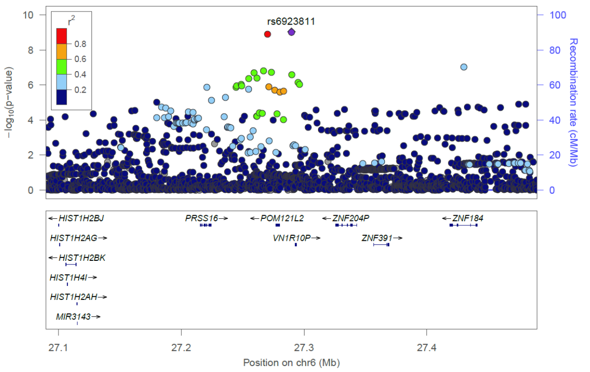


rs55960940

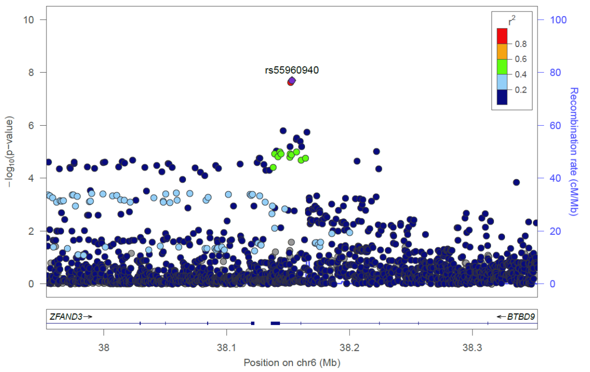

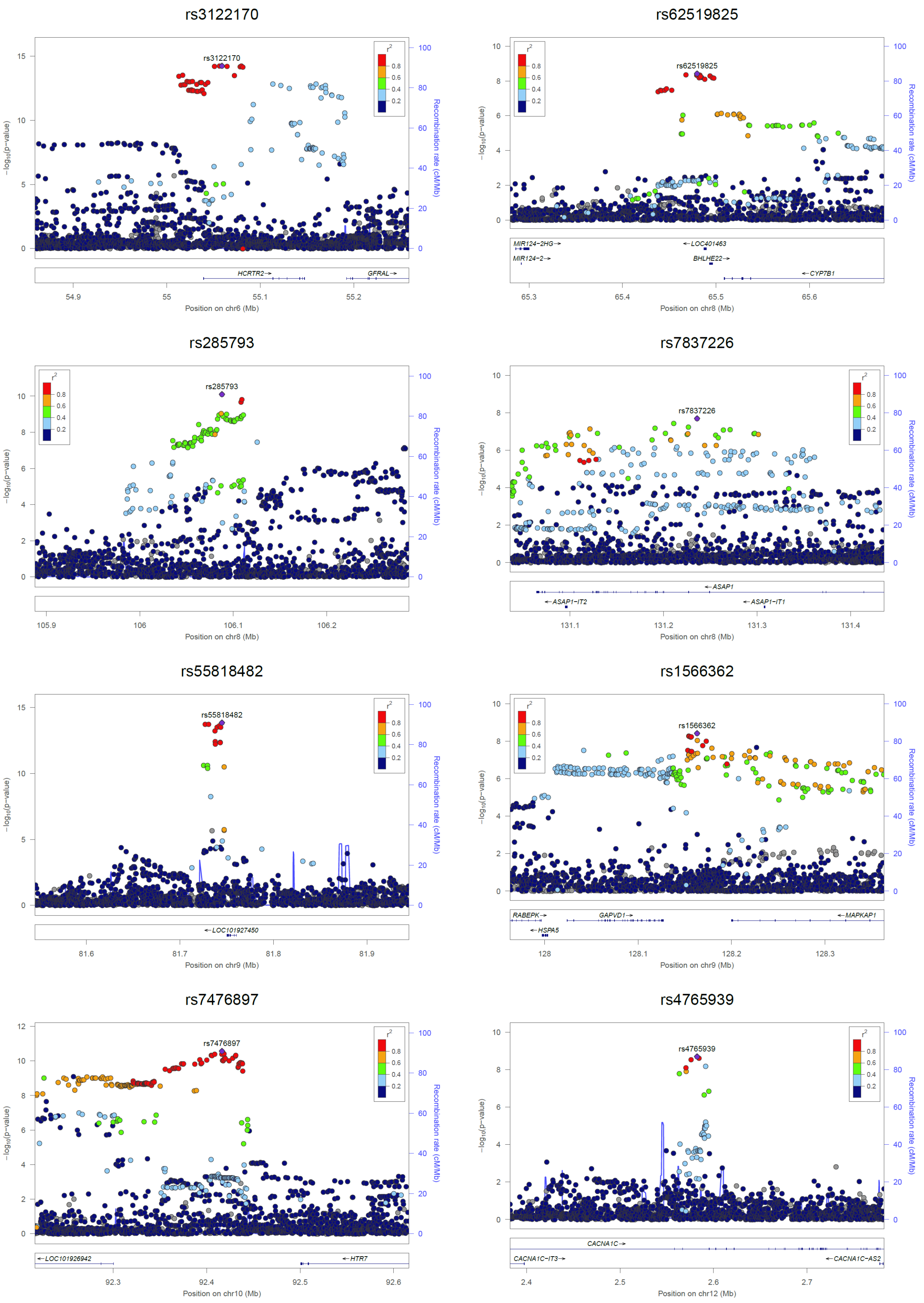
rs3122170
HCRTR2
GFRAL
Position on chr6 (Mb)
rs62519825
MIR124−2HG
MIR124−2
LOC401463
BHLHE22
CYP7B1
Position on chr8 (Mb)
rs285793
Position on chr8 (Mb)
rs7837226
ASAP1
ASAP1−IT2
ASAP1−IT1
Position on chr8 (Mb)
rs55818482
LOC101927450
Position on chr9 (Mb)
rs1566362
RABEPK
GAPVD1
MAPKAP1
HSPA5
Position on chr9 (Mb)
rs7476897
LOC101926942
HTR7
Position on chr10 (Mb)
rs4765939
CACNA1C
CACNA1C−IT3
CACNA1C−AS2
Position on chr12 (Mb)
−log10(p−value)
Recombination rate (cM/Mb)
r2

rs1846644

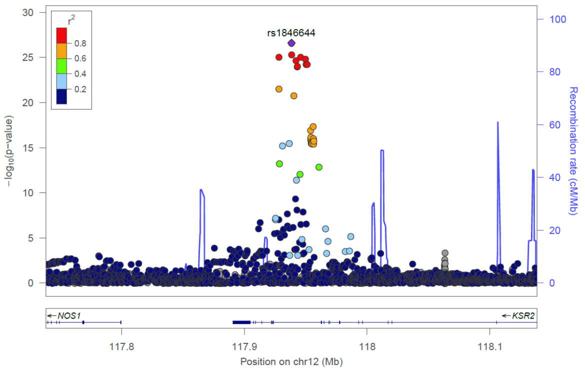


rs8015449

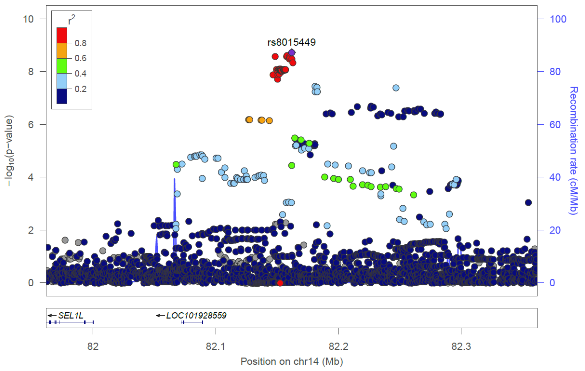


rs17131124

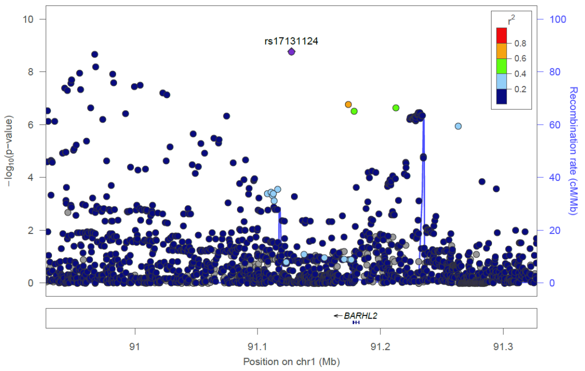


rs886114

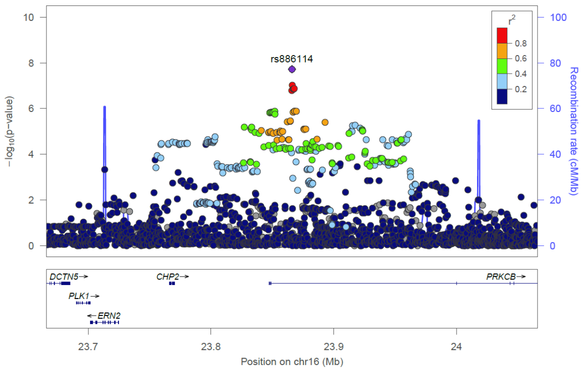


rs11078398

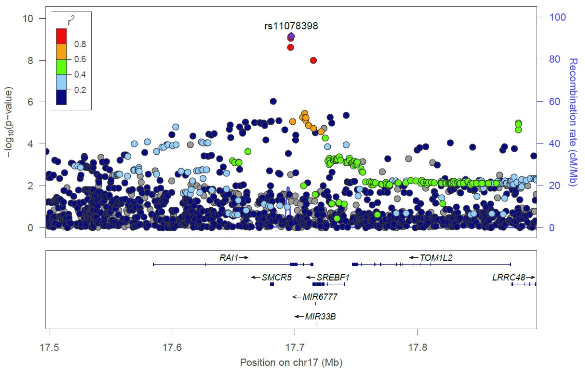


rs62066119

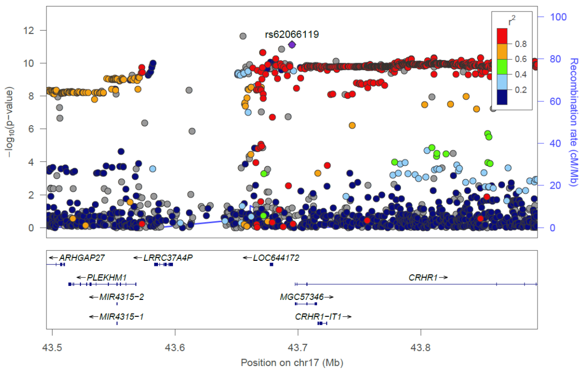


rs2048522

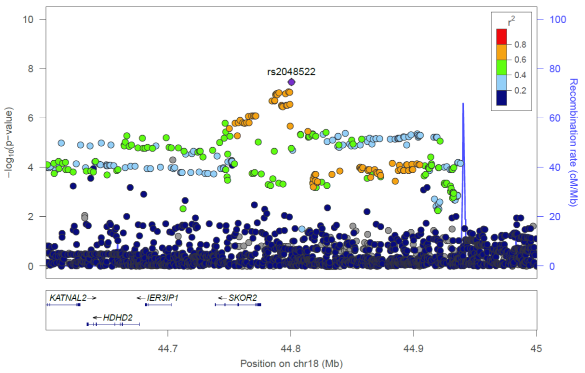


rs189568347

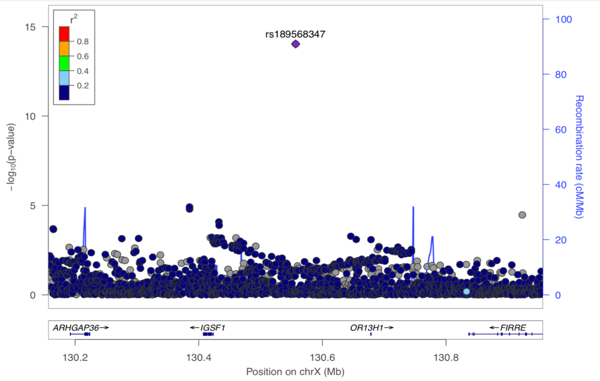

**Supplementary Figure 4.** Manhattan and QQ plots for genome-wide association analysis of self-reported daytime sleepiness adjusting for age, sex, **BMI**, genotyping array, top ten principal components and genetic relatedness matrix. Dashed line is genome-wide significant ($5\times10^{-8}$). Loci also significant without adjusting for BMI are highlighted in blue. Novel loci are highlighted in green. GC: Genomic Control; LDSR: LD Score Regression.

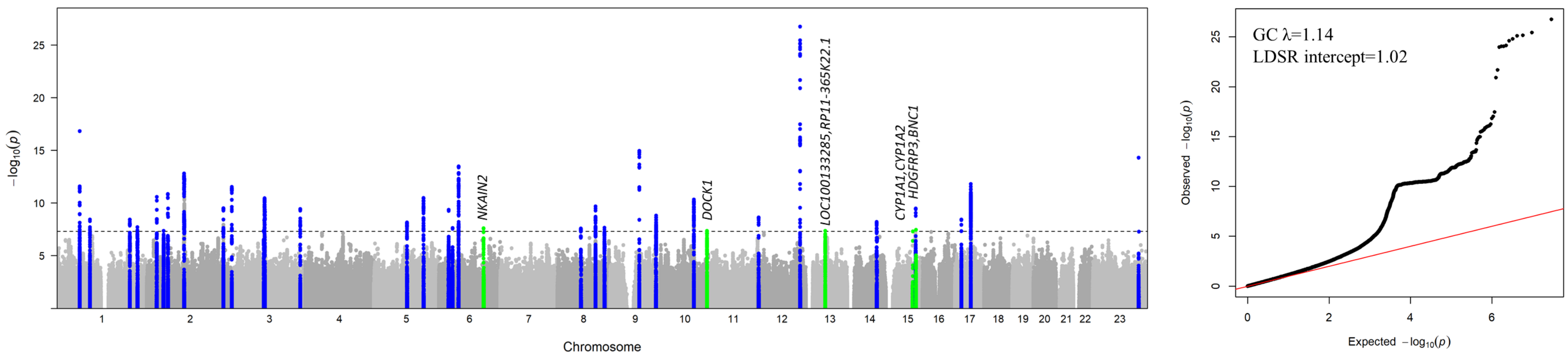

**Supplementary Figure 5.** Regional association plots of genome-wide significant loci ($5\times10^{-8}$) for self-reported daytime sleepiness adjusting for age, sex, **BMI**, genotyping array, top ten principal components and genetic relatedness matrix. Chromosomal position is indicated on the x-axis and –log10 p-values for each SNP (filled circles and squares) is indicated on the y-axis, with the lead SNP shown in purple (400kb window around lead SNP shown). Genes within the region are shown in the lower panel. The blue line indicates the recombination rate. Additional SNPs in the locus are colored according to linkage disequilibrium ($r^2$) with the lead SNP (estimated by LocusZoom based on the CEU HapMap haplotypes). Squares represent genotyped SNPs and circles represent imputed SNPs.

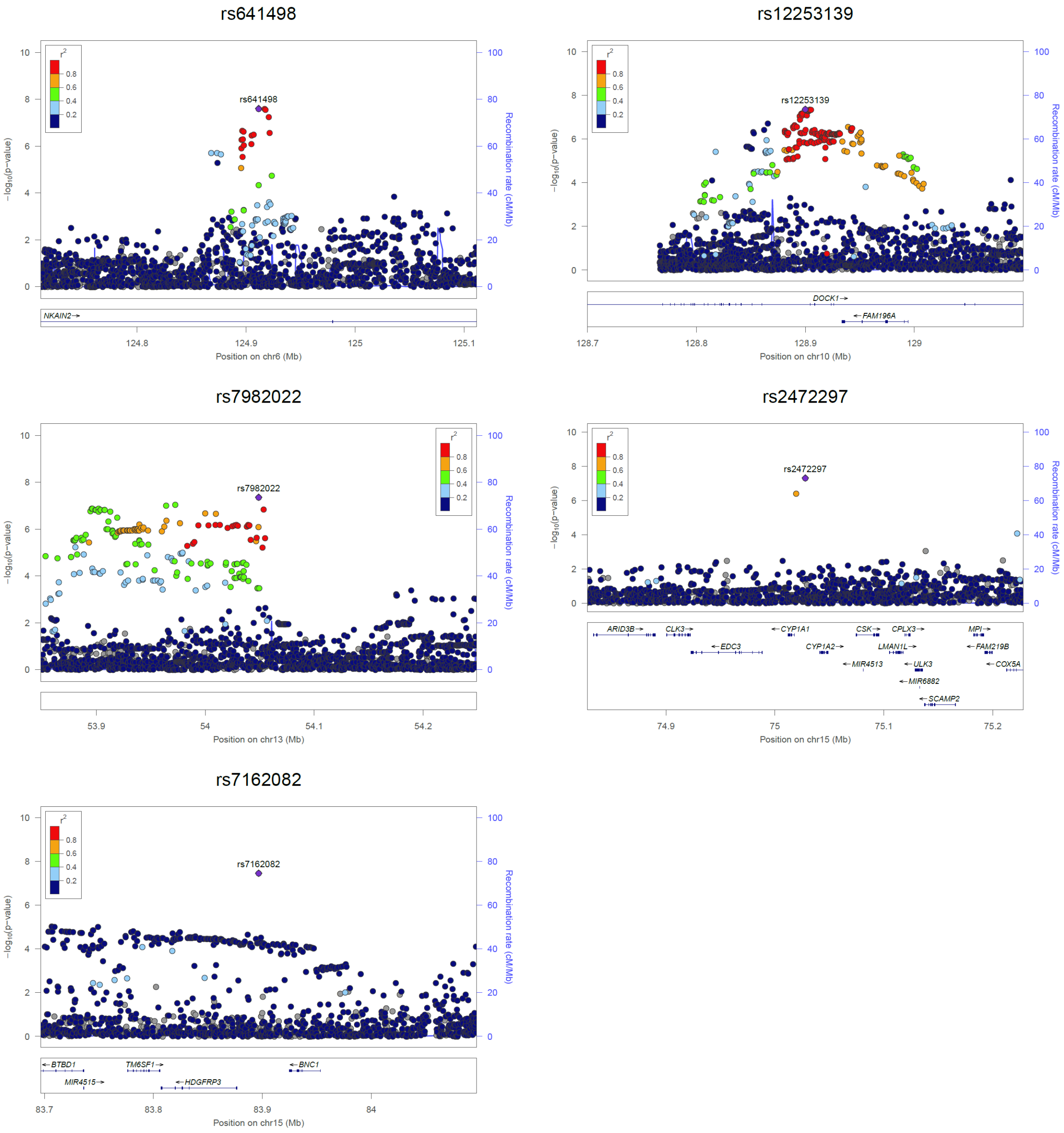

**Supplementary Figure 6**. Manhattan and QQ plots for a secondary genome-wide association analysis of self-reported daytime sleepiness adjusting for age, sex, genotyping array, and top ten principal components while excluding related individuals, shift workers, psychiatric medication users, and subjects with chronic and psychiatric illness. Dashed line is genome-wide significant ($5\times10^{-8}$). Loci also significant in primary model are highlighted in blue. Novel loci are highlighted in green. GC: Genomic Control; LDSR: LD Score Regression.

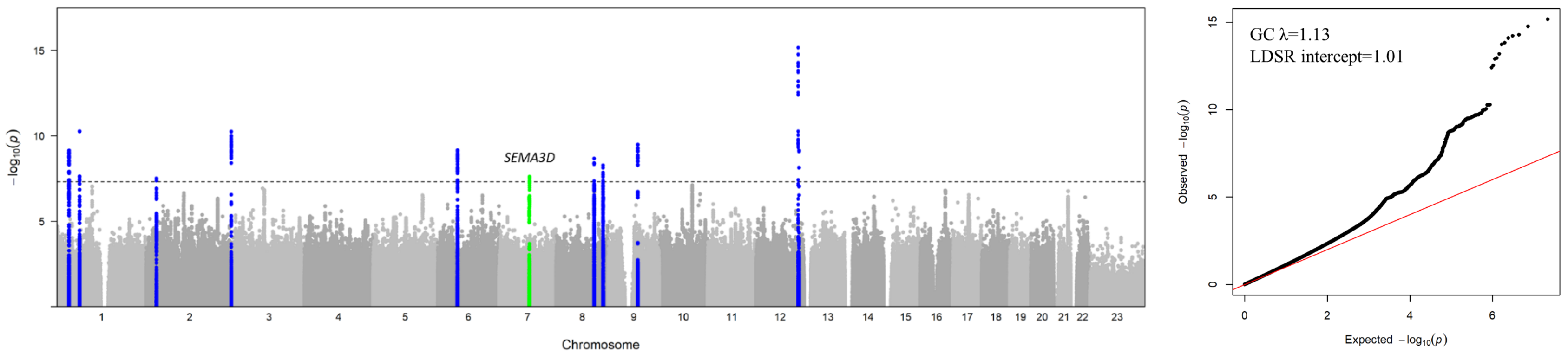

**Supplementary Figure 7.** Manhattan and QQ plots for genome-wide association analysis of sex-stratified self-reported daytime sleepiness adjusting for age, genotyping array, top ten principal components and genetic relatedness matrix. Dashed line is genome-wide significant ($5\times10^{-8}$). Loci also significant in pooled analysis are highlighted in blue. Novel sex-specific loci in females are highlighted in green. GC: Genomic Control; LDSR: LD Score Regression.

**A.** In males.

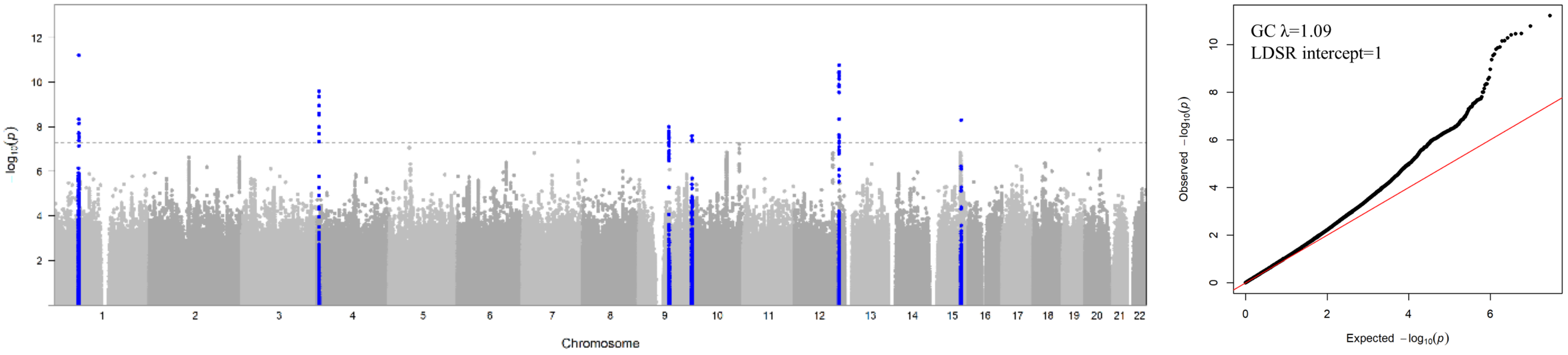


**B.** In females.

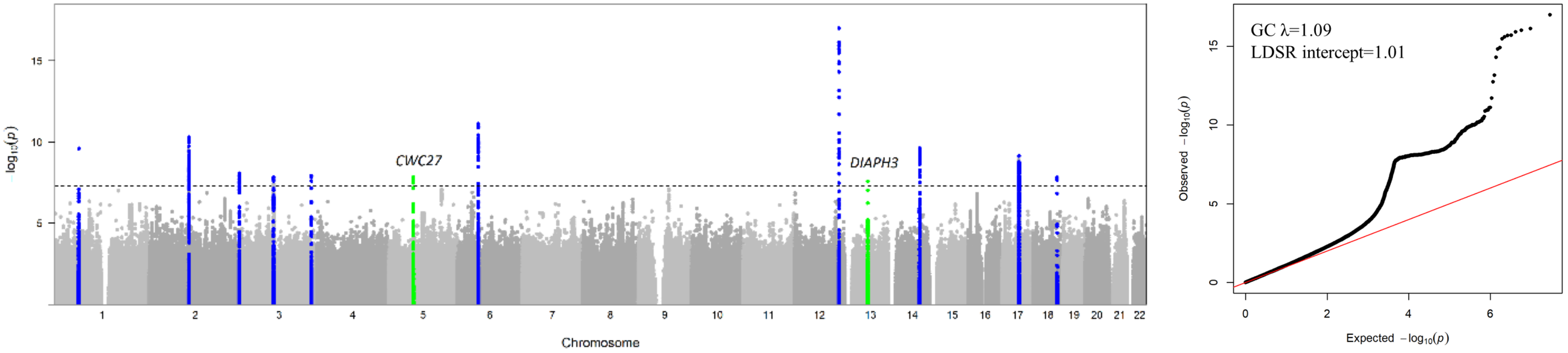

**Supplementary Figure 8.** Clustering silhouette plots of three iterations. Loci with negative silhouette coefficients were removed in the next iteration of clustering analysis.

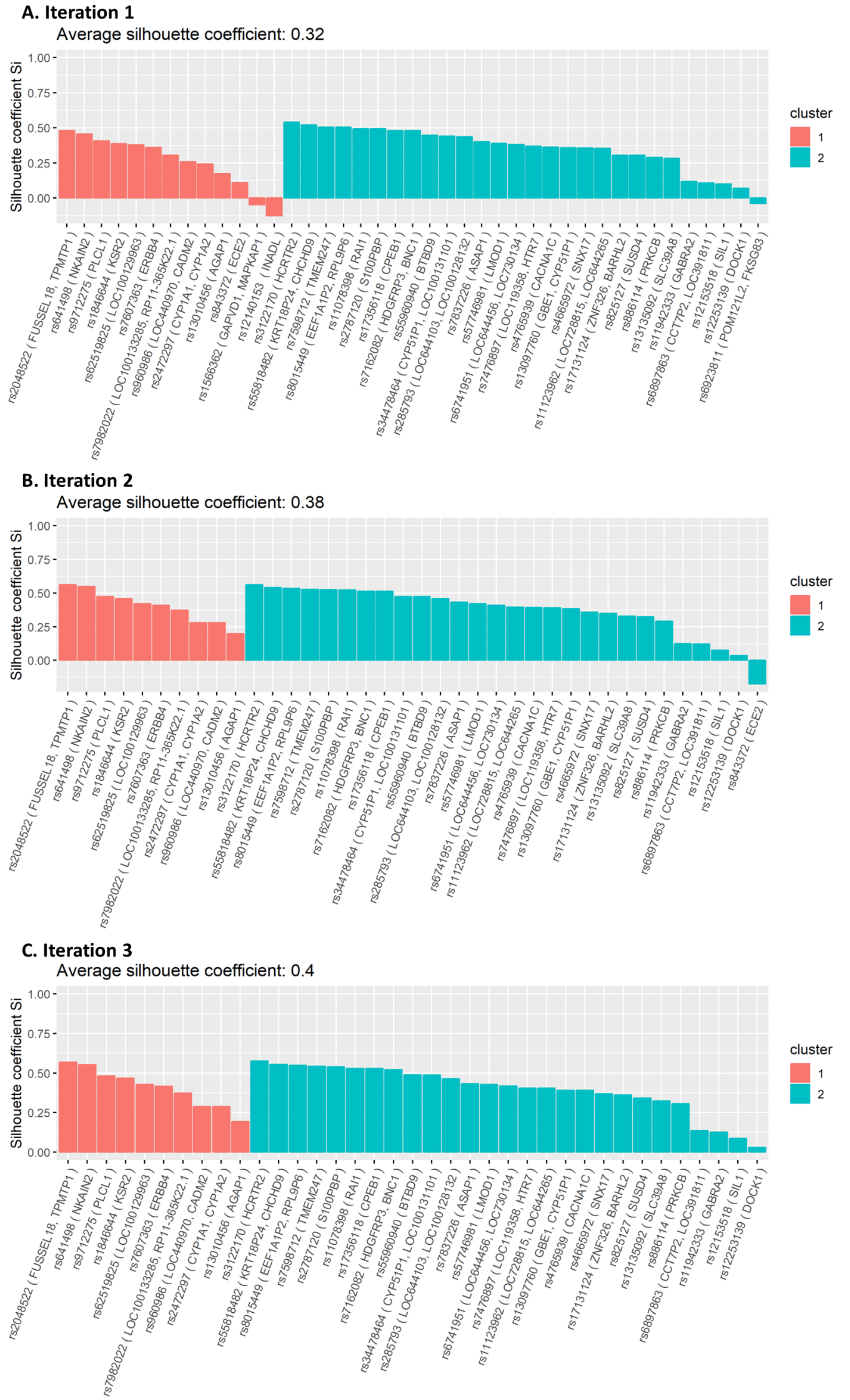

**Supplementary Figure 9.** Variants distribution of likely causal variants in the 42 genome-wide significant loci.

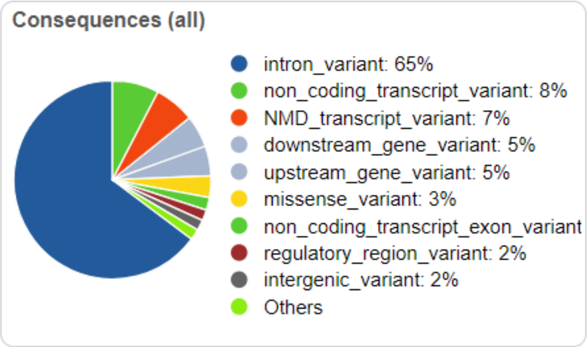

**Supplementary Figure 10.** Rs13135092 (SLC39A8) associated with brain structure and function traits in the UK Biobank using Oxford Brian Imagine Genetics (BIG) server.

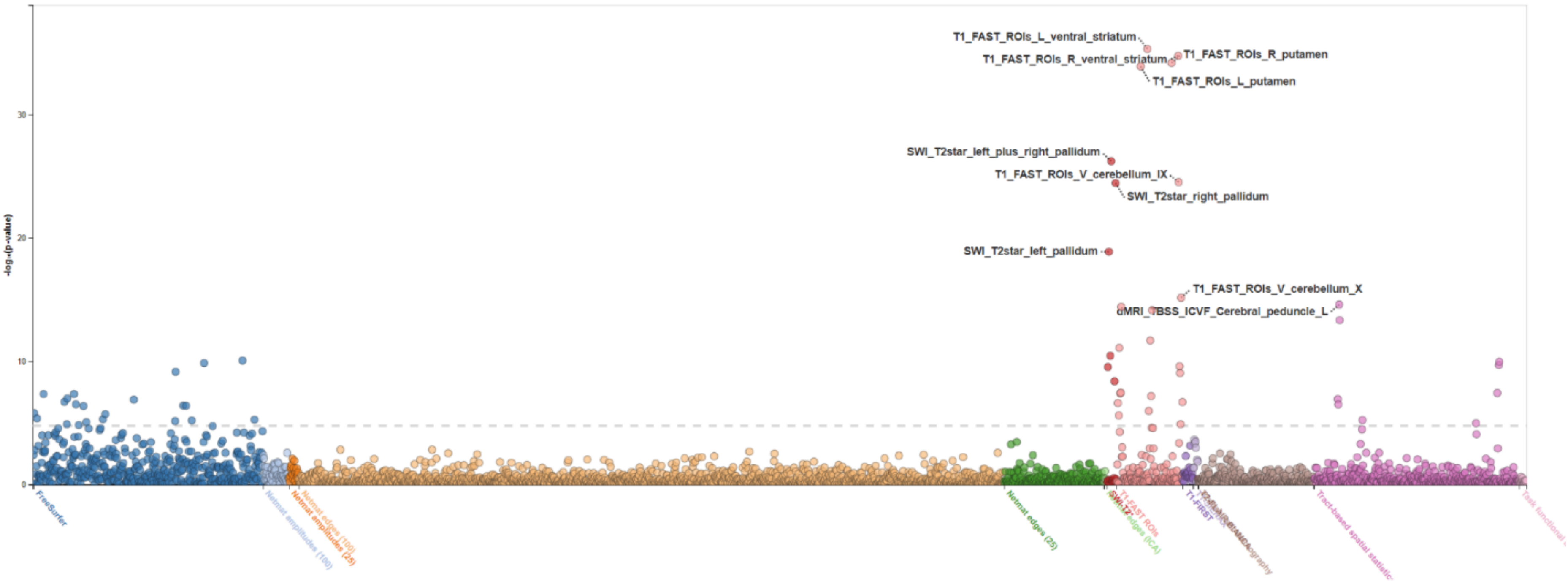

**Supplementary Figure 11.** Tissue expression analyses of genes overlapping A) all sleepiness loci, B) sleep propensity loci, and C) sleep fragmentation loci in GTEx RNA-seq data for 53 tissue types using MAGMA. Significant tissues ($P<9.43\times10^{-4}$) were highlighted in red.

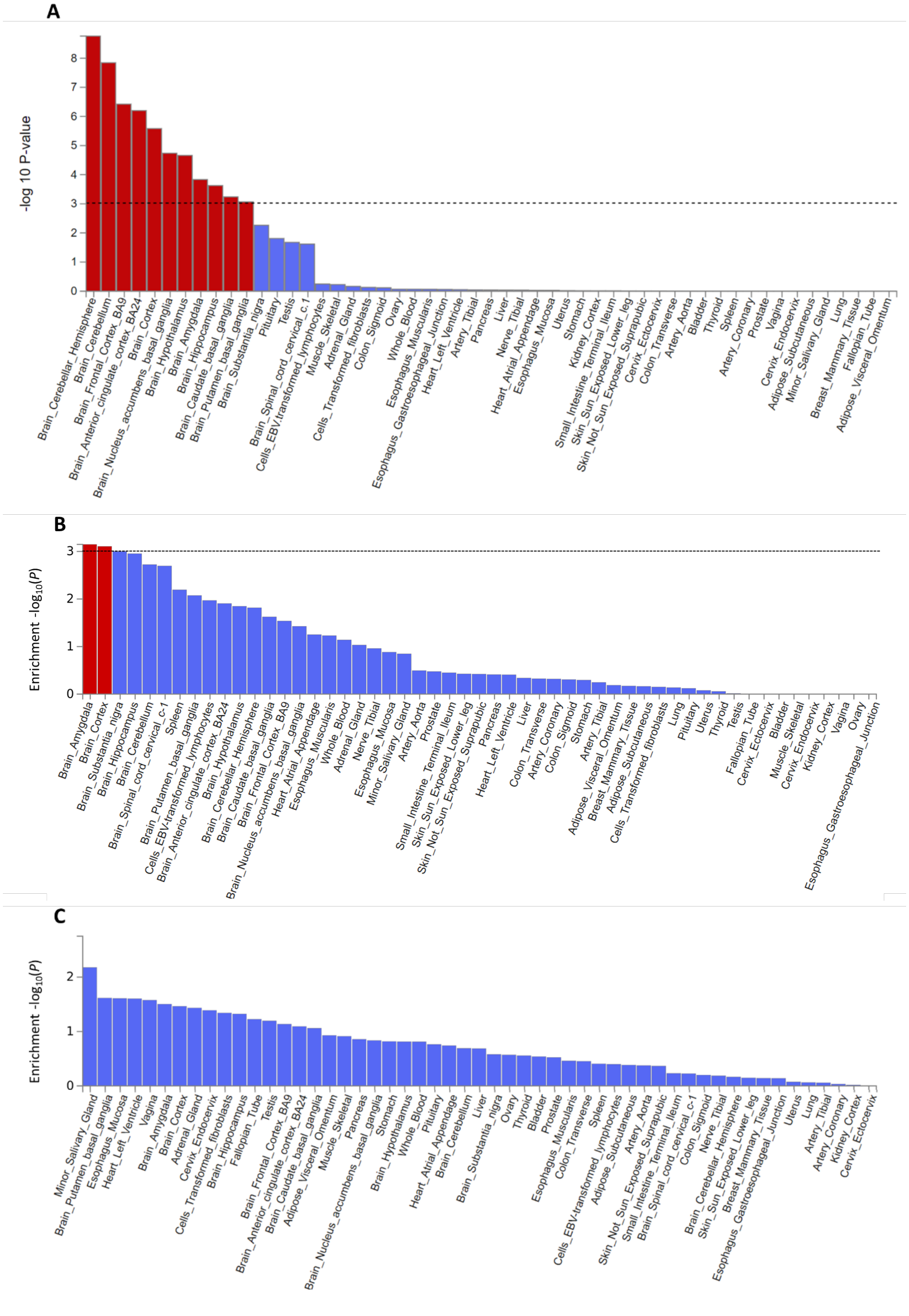

**Supplementary Figure 12.** Pathway analyses of genes overlapping with sleep propensity loci using FUMA.

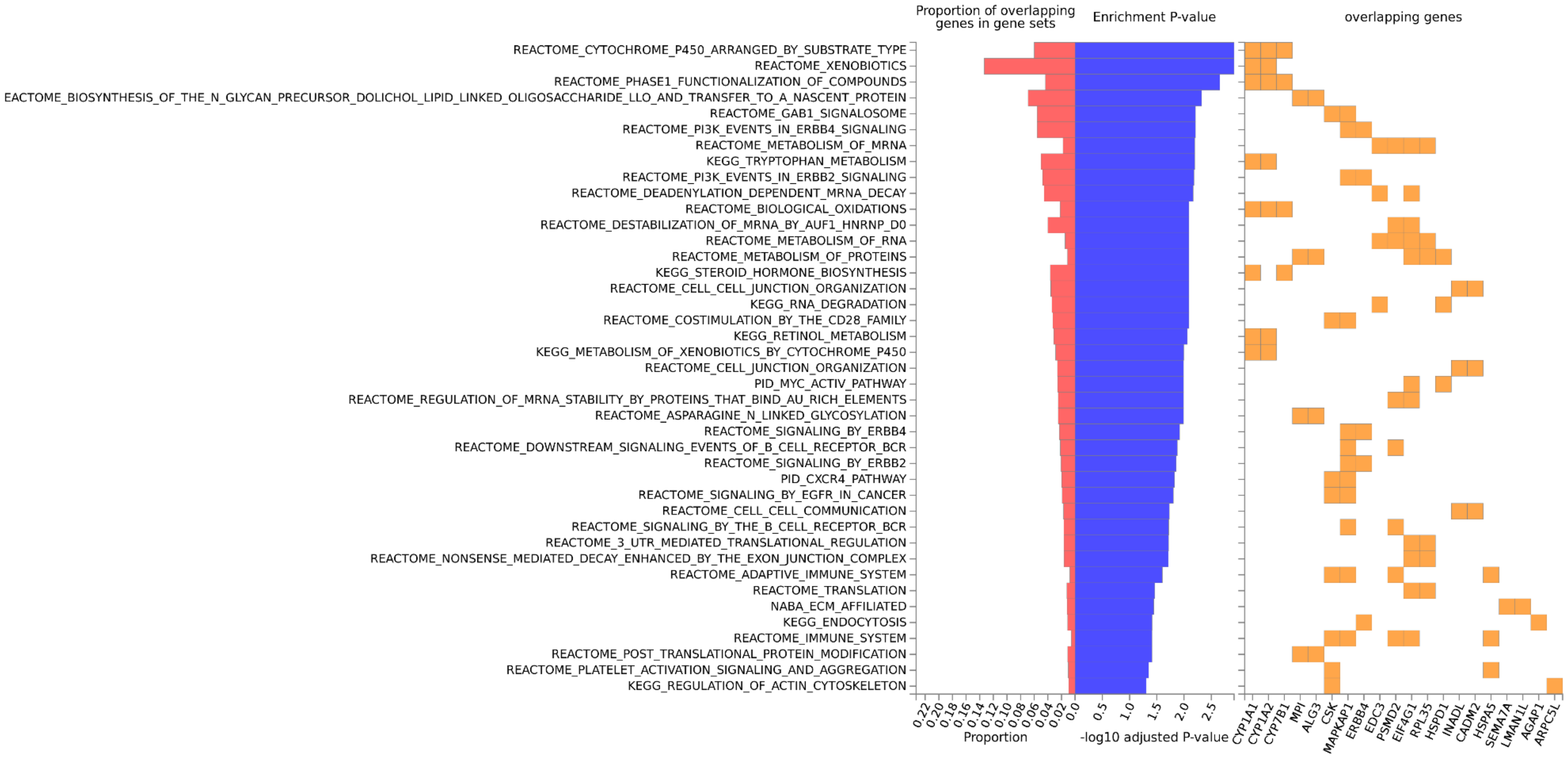

**Supplementary Figure 13.** Pathway analyses of genes overlapping with sleep fragmentation loci using FUMA.

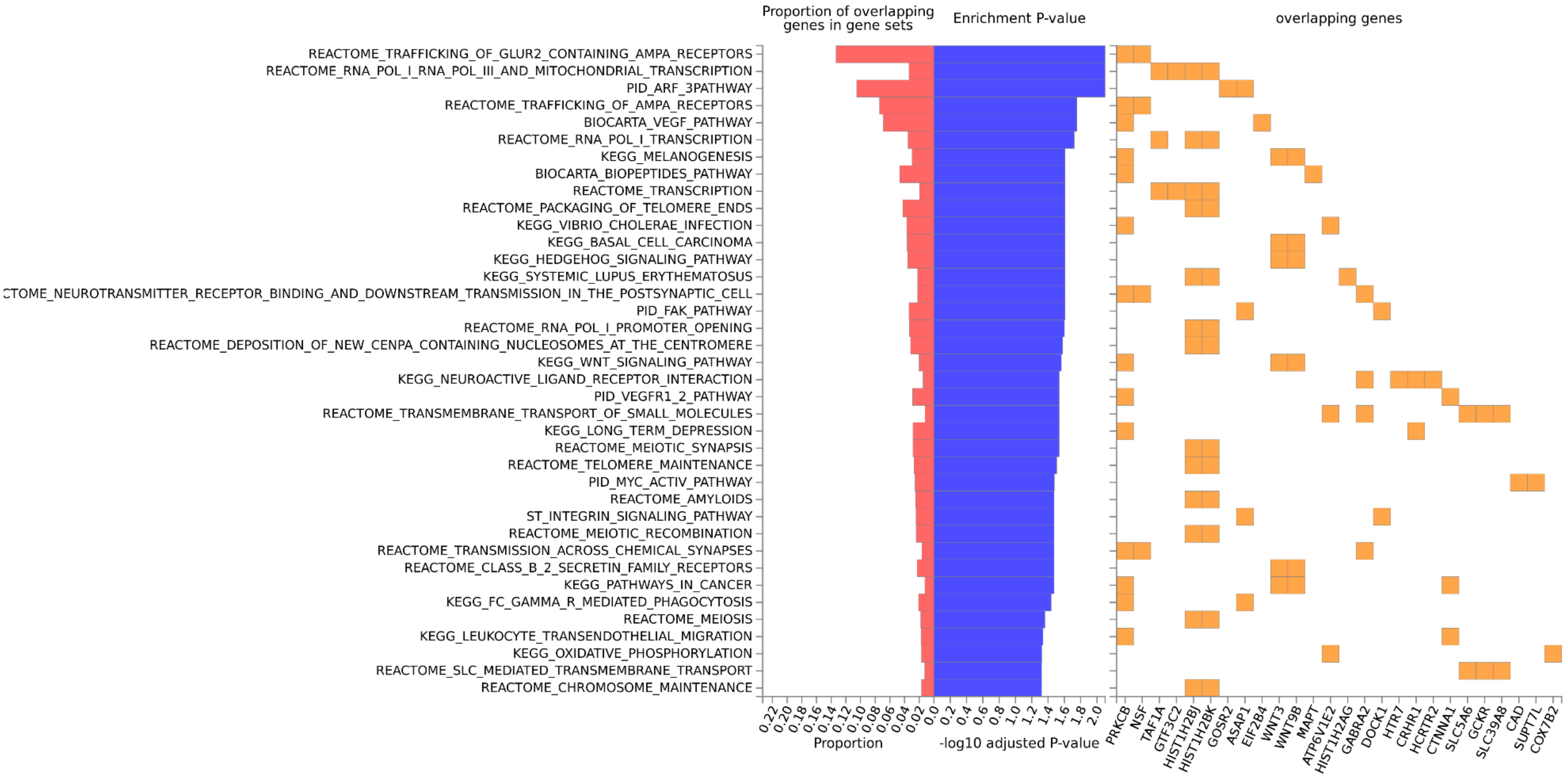

**Supplementary Table 1.** Descriptive characteristics of UBK subjects with self-reported daytime sleepiness.

| Characteristic | Self-reported Daytime Sleepiness | | | | | Spearman correlation to sleepiness | |
|---|---|---|---|---|---|---|---|
| | All | Never (N=347,285) | Sometimes (N=92,794) | Often (N=11,963) | All of the time (N=29) | rho | P-value |
| **Health status, N (%)** | | | | | | -0.06 | 0* |
| Without chronic and psychiatric diseases | 375944 | 292645 (77.8%) | 74748 (19.9%) | 8529 (2.3%) | 22 (0.006%) | | |
| With chronic and psychiatric diseases | 76127 | 54640 (71.8%) | 18046 (23.7%) | 3434 (4.5%) | 7 (0.009%) | | |
| **Male, N (%)** | 207764 | 155011 (74.6%) | 45721 (22%) | 6060 (2.9%) | 15 (0.007%) | -0.041 | $4.64\times10^{-166}$ |
| **Age (years), mean (SD)** | 57.27 (8.02) | 56.69 (8.06) | 59.23 (7.59) | 58.79 (7.63) | 57.64 (7.86) | 0.131 | 0* |
| **BMI (kg/m2), mean (SD)** | 27.4 (4.77) | 27.18 (4.62) | 27.99 (5.04) | 28.94 (5.86) | 29.71 (8.29) | 0.076 | 0* |
| Non-obese (BMI<30), N (%) | 344349 | 268757 (78%) | 66532 (19.3%) | 7789 (2.3%) | 20 (0.006%) | | |
| Obese (BMI≥30), N (%) | 109615 | 78528 (71.6%) | 26262 (24%) | 4174 (3.8%) | 9 (0.008%) | | |
| **Marital Status (have spouse/partner), N (%)** | 332262 | 256438 (77.2%) | 66975 (20.2%) | 7989 (2.4%) | 16 (0.005%) | -0.026 | $7.43\times10^{-65}$ |
| **Depression, N (%)** | 193504 | 143373 (74.1%) | 42636 (22%) | 6743 (3.5%) | 18 (0.009%) | 0.053 | $2.61\times10^{-275}$ |
| **Townsend Deprivation Index (SES), mean (SD)** | -1.47 (2.98) | -1.57 (2.92) | -1.24 (3.11) | -0.71 (3.35) | 0.53 (3.99) | 0.05 | $4.47\times10^{-249}$ |
| **Alcohol intake frequency, mean (SD)** | 2.86 (1.5) | 2.81 (1.47) | 2.98 (1.53) | 3.27 (1.65) | 2.9 (1.61) | 0.058 | 0* |
| **Coffee intake frequency, mean (SD)** | 2.17 (2) | 2.17 (1.98) | 2.17 (2.04) | 2.25 (2.28) | 2 (2.24) | -0.003 | 0.073 |
| **Tea intake frequency, mean (SD)** | 3.53 (2.73) | 3.49 (2.7) | 3.64 (2.79) | 3.79 (3.09) | 5.17 (4.83) | 0.024 | $9.30\times10^{-58}$ |
| **Smoking Status, N (%)** | | | | | | | |
| Current | 47285 | 35925 (76%) | 9496 (20.1%) | 1591 (3.4%) | 3 (0.006%) | 0.005 | 0.002 |
| Former | 160759 | 121160 (75.4%) | 34692 (21.6%) | 4414 (2.7%) | 13 (0.008%) | 0.021 | $8.68\times10^{-47}$ |
| Never/Rarely | 244019 | 189071 (77.5%) | 48235 (19.8%) | 5908 (2.4%) | 12 (0.005%) | -0.024 | $2.04\times10^{-58}$ |
| **Employment Status, N (%)** | | | | | | | |
| Employed | 258057 | 209532 (81.2%) | 43020 (16.7%) | 4925 (1.9%) | 11 (0.006%) | -0.126 | 0* |
| Retired | 156334 | 110596 (70.7%) | 40230 (25.7%) | 4859 (3.1%) | 9 (0.008%) | 0.099 | 0* |
| looking after home and/or family | 11862 | 9249 (78%) | 2268 (19.1%) | 306 (2.6%) | 0 (0%) | -0.005 | 0.0003 |
| unable to work because of sickness or disability | 14573 | 8331 (57.2%) | 4534 (31.1%) | 1465 (10.1%) | 7 (0.048%) | 0.086 | 0* |
| unemployed | 6429 | 4743 (73.8%) | 1410 (21.9%) | 215 (3.3%) | 1 (0.016%) | 0.007 | $2.88\times10^{-6}$ |
| Doing unpaid or voluntary work | 1958 | 1443 (73.7%) | 443 (22.6%) | 61 (3.1%) | 1 (0.051%) | 0.004 | 0.003 |
| full or part-time student | 931 | 733 (78.7%) | 167 (17.9%) | 26 (2.8%) | 0 (0%) | -0.002 | 0.108 |
| **Shift Worker, N (%)** | | | | | | 0.051 | $8.25\times10^{-147}$ |
| Never/Rarely | 215333 | 176789 (82.1%) | 34373 (16%) | 3814 (1.8%) | 7 (0.003%) | | |
| Sometimes | 18386 | 14456 (78.6%) | 3475 (18.9%) | 384 (2.1%) | 2 (0.011%) | | |
| Usually | 5069 | 3903 (77%) | 1014 (20%) | 122 (2.4%) | 0 (0%) | | |
| Always | 18795 | 14050 (74.8%) | 4049 (21.5%) | 584 (3.1%) | 2 (0.011%) | | |
| **Nap during day, N (%)** | | | | | | 0.344 | 0* |
| Never/Rarely | 255851 | 228079 (89.1%) | 26548 (10.4%) | 1219 (0.5%) | 5 (0.002%) | | |
| Sometimes | 172246 | 106788 (62%) | 59056 (34.3%) | 5393 (3.7%) | 9 (0.005%) | | |
| Usually | 23840 | 12346 (51.8%) | 7142 (30%) | 4338 (18.2%) | 14 (0.059%) | | |
| **Sleep Apnea, N (%)** | 5571 | 3001 (53.9%) | 1845 (33.1%) | 677 (12.2%) | 5 (0.09%) | 0.064 | 0* |
| **Narcolepsy, N (%)** | 12 | 3 (25%) | 4 (33.3%) | 5 (41.7%) | 0 (0%) | 0.007 | $1.14\times10^{-6}$ |
| **Sleep Duration (hours), mean (SD)** | 7.18 | 7.19 (1.03) | 7.14 (1.16) | 7.21 (1.61) | 7.42 (2.39) | -0.029 | $7.77\times10^{-85}$ |

| | | | | | | | |
|---|---|---|---|---|---|---|---|
| Short Sleep (<7), N (%) | 78393 | 56913 (72.6%) | 18399 (23.5%) | 2761 (3.5%) | 4 (0.005%) | 0.075 | 0* |
| Long Sleep (>8), N(%) | 25273 | 17850 (70.6%) | 5979 (23.7%) | 1320 (5.2%) | 7 (0.028%) | 0.065 | $2.89\times10^{-317}$ |
| **Insomnia, N (%)** | | | | | | 0.085 | 0* |
| Never/Rarely | 108357 | 88007 (81.2%) | 17962 (16.6%) | 2142 (2%) | 7 (0.006%) | | |
| Sometimes | 215752 | 166514 (77.2%) | 44731 (20.7%) | 3765 (1.7%) | 7 (0.003%) | | |
| Usually | 129270 | 92593 (71.6%) | 30029 (23.2%) | 6044 (4.7%) | 15 (0.012%) | | |
| **Chronotype, N (%)** | | | | | | 0.011 | $3.51\times10^{-13}$ |
| Definitely an 'evening' person | 36117 | 27044 (74.9%) | 7591 (21%) | 1349 (3.7%) | 5 (0.014%) | | |
| More a 'morning' than an 'evening' person | 145411 | 112527 (77.4%) | 29354 (20.2%) | 3214 (2.2%) | 5 (0.003%) | | |
| More an 'evening' than a 'moring' person | 115727 | 89605 (77.4%) | 23025 (19.9%) | 2793 (2.4%) | 4 (0.003%) | | |
| Definitely a 'morning' person | 108177 | 81106 (75%) | 23352 (21.6%) | 3341 (3.1%) | 9 (0.008%) | | |

*P-values smaller than machine epsilon (the relative error due to rounding in computer floating point arithmetic) were shown as 0.

**Supplementary Table 2.** Descriptive characteristics of UBK subjects with accelerometry measurements.

| | Self-reported Daytime Sleepiness | | | | | Spearman correlation to sleepiness | |
|---|---|---|---|---|---|---|---|
| **Characteristic** | **All (N=85,388)** | **Never (N=67,168)** | **Sometimes (N=16,312)** | **Often (N=1,905)** | **All of the time (N=3)** | **rho** | **P-value** |
| Sex, male (n,%) | 37361 | 28487 (76.2%) | 7950 (21.3%) | 922 (2.5%) | 2 (0.005%) | 0.049 | $3.25\times10^{-47}$ |
| Age (years), mean (SD) | 62.52 (7.8) | 62.02 (7.85) | 64.42 (7.32) | 64.15 (7.19) | 63.61 (7.71) | 0.118 | $2.29\times10^{-261}$ |
| BMI (kg/m2), mean (SD) | 26.69 (4.51) | 26.55 (4.41) | 27.13 (4.76) | 27.78 (5.55) | 21.59 (3.29) | 0.061 | $7.61\times10^{-72}$ |
| Midpoint of 5-hour daily period of minimum activity (L5 timing) (h after previous midnight), mean (SD) | 27.32 (1.06) | 27.3 (1.05) | 27.36 (1.08) | 27.36 (1.26) | 26.83 (0.93) | 0.020 | $9.95\times10^{-9}$ |
| Midpoint of 10-hour daily period of maximum activity (M10 timing) (h after previous midday), mean (SD) | 13.7 (1.21) | 13.72 (1.2) | 13.62 (1.22) | 13.63 (1.34) | 14.1 (1.35) | -0.032 | $6.09\times10^{-21}$ |
| Sleep duration (minutes), mean (SD) | 438.06 (51.65) | 439.77 (50.91) | 432.5 (53.42) | 425.22 (56.87) | 412.04 (40.62) | -0.067 | $5.48\times10^{-85}$ |
| Sleep duration standard deviation (minutes), mean (SD) | 55.83 (34.1) | 55.76 (34.33) | 55.79 (33.2) | 58.39 (33.32) | 61.35 (40.64) | 0.007 | 0.0531 |
| Sleep Midpoint (h after previous midnight), mean (SD) | 26.99 (0.91) | 26.99 (0.9) | 26.99 (0.93) | 26.94 (1.06) | 26.81 (1.2) | -0.004 | 0.2998 |
| Sleep efficiency %, mean (SD) | 76.19 (7.18) | 76.54 (7.04) | 75.02 (7.42) | 73.72 (8.02) | 74.01 (5.88) | -0.098 | $2.93\times10^{-181}$ |
| Number of sleep bouts (n), mean (SD) | 17.25 (3.59) | 17.3 (3.59) | 17.11 (3.59) | 16.97 (3.59) | 16.43 (2.76) | -0.023 | $1.53\times10^{-11}$ |
| Daytime Inactivity Duration (minutes), mean (SD) | 58.1 (40.52) | 56.53 (39.77) | 63.1 (41.75) | 70.54 (49.41) | 46.8 (29.92) | 0.079 | $6.83\times10^{-117}$ |

**Supplementary Table 3**. Genome-wide significant (P<5×10-8) loci associated with self-reported daytime sleepiness with and without adjusting for BMI in subjects of European ancestry in the UK Biobank.

| SNP | Chr:position (NCBI Build 37) | Nearest Gene(s) | Alleles (E/A) | EAF | INFO | Without adjusting for BMI | | Adjusting for BMI | |
|---|---|---|---|---|---|---|---|---|---|
| | | | | | | Beta (SE) | P-value | β | P-value |
| rs2787120 | 1:33306297 | *S100PBP* | A/G | 0.83 | 1 | 0.008 (0.001) | $2.00\times10^{-8}$ | 0.007 (0.001) | $3.10\times10^{-7}$ |
| rs12140153* | 1: 62579891 | *PATJ* | G/T | 0.9 | 0.95 | 0.017 (0.002) | $2.80\times10^{-20}$ | 0.015 (0.002) | $1.50\times10^{-17}$ |
| rs17131124 | 1:91127548 | *ZNF326, BARHL2* | C/G | 0.91 | 0.97 | -0.011 (0.002) | $1.70\times10^{-9}$ | -0.011 (0.002) | $5.40\times10^{-9}$ |
| rs57746981 | 1:201885234 | *LMOD1* | C/T | 0.64 | 1 | 0.007 (0.001) | $2.20\times10^{-10}$ | 0.006 (0.001) | $3.80\times10^{-9}$ |
| rs825127 | 1:223506788 | *SUSD4* | T/G | 0.53 | 0.99 | 0.006 (0.001) | $9.50\times10^{-9}$ | 0.006 (0.001) | $2.00\times10^{-8}$ |
| rs4665972 | 2:27598097 | *SNX17* | T/C | 0.39 | 0.99 | 0.007 (0.001) | $3.90\times10^{-10}$ | 0.007 (0.001) | $2.70\times10^{-11}$ |
| rs7598712 | 2:46660452 | *TMEM247* | G/T | 0.56 | 0.99 | 0.006 (0.001) | $2.20\times10^{-8}$ | 0.006 (0.001) | $4.40\times10^{-8}$ |
| rs6741951 | 2:58959112 | *LOC644456, LOC730134* | G/A | 0.71 | 0.99 | 0.007 (0.001) | $2.70\times10^{-9}$ | 0.008 (0.001) | $1.40\times10^{-11}$ |
| rs11123962 | 2:104157011 | *LOC728815, LOC644265* | T/G | 0.55 | 1 | -0.008 (0.001) | $7.50\times10^{-15}$ | -0.008 (0.001) | $1.60\times10^{-13}$ |
| rs9712275 | 2:198907143 | *PLCL1* | C/T | 0.49 | 0.99 | -0.006 (0.001) | $1.30\times10^{-8}$ | -0.005 (0.001) | $2.20\times10^{-7}$ |
| rs7607363 | 2:213402705 | *ERBB4* | A/G | 0.56 | 1 | -0.006 (0.001) | $8.00\times10^{-9}$ | -0.007 (0.001) | $3.10\times10^{-10}$ |
| rs13010456 | 2:236792801 | *AGAP1* | A/G | 0.6 | 0.99 | 0.008 (0.001) | $2.10\times10^{-13}$ | 0.007 (0.001) | $2.90\times10^{-12}$ |
| rs13097760 | 3:82823561 | *GBE1, CYP51P1* | A/C | 0.64 | 0.99 | -0.006 (0.001) | $3.20\times10^{-8}$ | -0.006 (0.001) | $4.10\times10^{-8}$ |
| rs34478464 | 3:84228726 | *CYP51P1, LOC100131101* | C/T | 0.81 | 1 | -0.009 (0.001) | $5.80\times10^{-11}$ | -0.008 (0.001) | $1.70\times10^{-10}$ |
| rs960986 | 3:85519305 | *LOC440970, CADM2* | C/T | 0.64 | 1 | 0.007 (0.001) | $1.50\times10^{-11}$ | 0.007 (0.001) | $4.20\times10^{-10}$ |
| rs843372 | 3:183996213 | *ECE2* | C/T | 0.23 | 1 | 0.008 (0.001) | $2.20\times10^{-11}$ | 0.008 (0.001) | $3.80\times10^{-10}$ |
| rs11942333 | 4:46389486 | *GABRA2* | G/A | 0.68 | 0.99 | -0.006 (0.001) | $3.80\times10^{-8}$ | -0.006 (0.001) | $6.80\times10^{-8}$ |
| rs13135092 | 4:103198082 | *SLC39A8* | A/G | 0.92 | 0.99 | -0.010 (0.002) | $3.10\times10^{-8}$ | -0.009 (0.002) | $3.40\times10^{-6}$ |
| rs6897863 | 5:92512481 | *CCT7P2, LOC391811* | A/C | 0.58 | 0.99 | 0.006 (0.001) | $7.60\times10^{-10}$ | 0.006 (0.001) | $6.90\times10^{-9}$ |
| rs12153518 | 5:138501494 | *SIL1* | A/C | 0.47 | 1 | 0.007 (0.001) | $6.80\times10^{-11}$ | 0.007 (0.001) | $3.30\times10^{-11}$ |
| rs6923811 | 6:27289776 | *POM121L2, FKSG83* | T/C | 0.68 | 1 | 0.007 (0.001) | $9.10\times10^{-10}$ | 0.007 (0.001) | $4.50\times10^{-10}$ |
| rs55960940 | 6:38153146 | *BTBD9* | T/C | 0.82 | 0.99 | 0.008 (0.001) | $2.00\times10^{-8}$ | 0.008 (0.001) | $2.30\times10^{-8}$ |
| rs3122170 | 6:55058998 | *HCRTR2* | C/A | 0.23 | 0.99 | 0.010 (0.001) | $5.60\times10^{-15}$ | 0.009 (0.001) | $3.40\times10^{-14}$ |
| rs62519825 | 8:65479707 | *LOC100129963* | T/C | 0.89 | 1 | -0.009 (0.002) | $3.80\times10^{-9}$ | -0.009 (0.002) | $2.60\times10^{-8}$ |
| rs285793 | 8:106087862 | *LOC644103, LOC100128132* | G/A | 0.46 | 0.99 | 0.007 (0.001) | $7.90\times10^{-11}$ | 0.007 (0.001) | $2.10\times10^{-10}$ |
| rs7837226 | 8:131235895 | *ASAP1* | A/G | 0.47 | 1 | -0.006 (0.001) | $2.00\times10^{-8}$ | -0.006 (0.001) | $2.20\times10^{-8}$ |
| rs55818482 | 9:81744922 | *KRT18P24, CHCHD9* | T/C | 0.79 | 0.99 | -0.010 (0.001) | $1.40\times10^{-14}$ | -0.010 (0.001) | $1.20\times10^{-15}$ |
| rs1566362 | 9:128163132 | *GAPVD1, MAPKAP1* | T/C | 0.63 | 0.99 | 0.006 (0.001) | $3.80\times10^{-9}$ | 0.006 (0.001) | $1.60\times10^{-9}$ |
| rs7476897 | 10:92416402 | *LOC119358, HTR7* | G/A | 0.68 | 1 | 0.007 (0.001) | $2.70\times10^{-11}$ | 0.007 (0.001) | $4.60\times10^{-11}$ |
| rs4765939 | 12:2582397 | *CACNA1C* | G/C | 0.58 | 0.99 | -0.006 (0.001) | $2.00\times10^{-9}$ | -0.006 (0.001) | $2.30\times10^{-9}$ |
| rs1846644 | 12:117938380 | *KSR2* | T/C | 0.59 | 1 | -0.011 (0.001) | $2.50\times10^{-27}$ | -0.011 (0.001) | $1.80\times10^{-27}$ |
| rs8015449 | 14:82161860 | *EEF1A1P2, RPL9P6* | A/G | 0.54 | 1 | 0.006 (0.001) | $1.90\times10^{-9}$ | 0.006 (0.001) | $6.20\times10^{-9}$ |
| rs17356118 | 15:83237899 | *CPEB1* | A/G | 0.77 | 1 | -0.008 (0.001) | $2.60\times10^{-10}$ | -0.008 (0.001) | $3.20\times10^{-10}$ |
| rs886114 | 16:23865986 | *PRKCB* | C/T | 0.36 | 0.99 | 0.006 (0.001) | $1.90\times10^{-8}$ | 0.006 (0.001) | $5.40\times10^{-8}$ |
| rs11078398† | 17:17697099 | *RAI1* | G/A | 0.74 | 0.91 | 0.008 (0.001) | $7.10\times10^{-10}$ | 0.007 (0.001) | $3.80\times10^{-9}$ |
| rs62066119 | 17:43695197 | *LOC644191, MGC57346* | C/T | 0.75 | 0.95 | 0.008 (0.001) | $7.30\times10^{-12}$ | 0.008 (0.001) | $2.80\times10^{-12}$ |
| rs2048522 | 18:44800515 | *FUSSEL18, TPMTP1* | A/T | 0.57 | 0.98 | 0.006 (0.001) | $3.50\times10^{-8}$ | 0.005 (0.001) | $2.20\times10^{-7}$ |
| rs189568347‡ | X:130556696 | *IGSF1* | C/G | 0.99 | 0.91 | -0.046 (0.006) | $9.40\times10^{-15}$ | -0.046 (0.006) | $5.10\times10^{-15}$ |
| rs641498 | 6:124911565 | *NKAIN2* | A/G | 0.39 | 0.99 | -0.005 (0.001) | $2.70\times10^{-7}$ | -0.006 (0.001) | $2.50\times10^{-8}$ |
| rs12253139 | 10:128899947 | *DOCK1* | T/C | 0.88 | 1 | -0.008 (0.002) | $1.20\times10^{-7}$ | -0.009 (0.002) | $4.40\times10^{-8}$ |
| rs7982022 | 13:54049003 | *LOC100133285, RP11-365K22.1* | G/A | 0.56 | 0.96 | -0.006 (0.001) | $1.60\times10^{-7}$ | -0.006 (0.001) | $4.40\times10^{-8}$ |
| rs2472297 | 15:75027880 | *CYP1A1, CYP1A2* | C/T | 0.74 | 1 | 0.006 (0.001) | $6.80\times10^{-7}$ | 0.006 (0.001) | $4.80\times10^{-8}$ |
| rs7162082 | 15:83896608 | *HDGFRP3, BNC1* | C/T | 0.8 | 1 | 0.007 (0.001) | $6.60\times10^{-8}$ | 0.007 (0.001) | $3.50\times10^{-8}$ |

*rs12140153 is a missense variant. †rs11078398 is a nonsynonmous variant. ‡rs189568347 locus is driven by a single rare variant therefore not reported as main finding.

Significant loci with P<5×10-8 were highlighted in red.

**Supplementary Table 4.** Associations of previously reported genetic variants for daytime sleepiness and other related traits in the UK Biobank.

| SNP | Gene | Chr:position (NCBI Build 37) | Alleles (E/A) | EAF | INFO | BETA (SE) | P | Reference | Original Trait |
|---|---|---|---|---|---|---|---|---|---|
| rs1986116 | *WNCG* | 14:77513844 | T/C | 0.239 | 0.99 | 0.001 (0.001) | 0.24 | PMID:23728906 | Sleep quality |
| rs1823068 | *PDE4D* | 5:58676049 | A/G | 0.857 | 0.98 | -0.001 (0.001) | 0.72 | PMID:17903308 | Sleepiness |
| rs2858884 | *HLA-DQ2* | 6:32700083 | A/C | 0.776 | 1.00 | 0.001 (0.001) | 0.31 | PMID:20711174 | Narcolepsy |
| rs1154155 | *TRA* | 14:23002684 | T/G | 0.849 | 1.00 | 0.0004 (0.001) | 0.77 | PMID:19412176, bioRxiv:169623, bioRxiv:373555 | Narcolepsy |
| rs12425451 | *TEAD4* | 12:3164923 | C/T | 0.279 | 0.97 | -0.001 (0.001) | 0.63 | PMID:23496005 | Age at onset of cataplexy in narcolepsy |
| rs16966122 | *THEG5* | 19:32158464 | A/G | 0.832 | 1.00 | 0.0001 (0.001) | 0.91 | PMID:23496005 | ESS |
| rs2859998 | *UBXN2B* | 8:59324162 | G/A | 0.700 | 1.00 | 0.001 (0.001) | 0.26 | PMID:23496005 | Age at onset of EDS in narcolepsy |
| rs5770917 | *CPT1B* | 22:51017353 | T/C | 0.952 | 1.00 | 0.0002 (0.002) | 0.97 | PMID:18820697 | Narcolepsy |
| rs11854769 | *SPRED1* | 15:38502243 | C/T | 0.752 | 0.99 | 0.001 (0.001) | 0.57 | PMID:23646285 | HLA negative essential hypersomnia |
| rs10988217 | *CRAT* | 9:131888116 | A/G | 0.396 | 0.98 | 0.002 (0.001) | 0.097 | PMID:23646285 | HLA negative essential hypersomnia |
| rs16826005 | *NCKAP1* | 2:134266001 | A/G | 0.960 | 1.00 | -0.001 (0.003) | 0.63 | PMID:23646285 | HLA negative essential hypersomnia |
| rs7553711 | *GOT2L2, TNFSF4* | 1:173000000 | C/T | 0.285 | 0.98 | 0.001 (0.001) | 0.21 | bioRxiv:169623 | Narcolepsy |
| rs10915020 | *C1orf94, GJB5* | 1:35157716 | A/T | 0.087 | 1.00 | 0.002 (0.002) | 0.4 | bioRxiv:169623 | Narcolepsy |
| rs10995245 | *ZNF365* | 10:64391375 | A/G | 0.349 | 0.99 | 0.001 (0.001) | 0.5 | bioRxiv:169623, bioRxiv:373555 | Narcolepsy |
| rs34593439 | *CTSH* | 15:79234957 | A/G | 0.104 | 0.99 | 0.001 (0.002) | 0.45 | bioRxiv:169623, bioRxiv:373555 | Narcolepsy |
| rs2834168 | *IL10RB, IFNAR1* | 21:34650791 | A/G | 0.694 | 1.00 | 0.003 (0.001) | 0.021 | bioRxiv:169623, bioRxiv:373555 | Narcolepsy |
| rs13383830 | CD207 (Langerin) | 2:71058306 | T/C | 0.948 | 1.00 | 0.002 (0.002) | 0.29 | bioRxiv:373555 | Narcolepsy |
| rs75674288 | ZFAND2 | 7:1195322 | A/C | 0.995 | 1.00 | -0.005 (0.007) | 0.49 | bioRxiv:373555 | Narcolepsy |
| rs1008599 | TRB | 7:142038782 | A/G | 0.557 | 0.96 | 0.0002 (0.001) | 0.83 | bioRxiv:373555 | Narcolepsy |
| rs35947132 | PRF1 | 10:72360387 | G/A | 0.960 | 0.99 | 0.006 (0.003) | 0.033 | bioRxiv:373555 | Narcolepsy |
| rs73536079 | *AR, OPHN1* | X:67154206 | C/G | 0.999 | 0.96 | -0.092 (0.033) | 0.006 | PMID:27992416* | Sleepiness |
| rs182765975 | *ROBO1* | 3:78538431 | G/T | 0.996 | 0.87 | -0.023 (0.008) | 0.007 | PMID:27992416* | Sleepiness |
| rs142261172† | *TMEM132B* | 12:126049981 | G/A | 0.995 | 0.93 | -0.016 (0.008) | 0.042 | PMID:27992416* | Sleepiness |

*Previous UK Biobank sleep study of 111,975 individuals. Samples overlapped with the current study.
†Adjusted for BMI.

**Supplementary Table 5.** Stratified analysis of significant loci on autosomes by obesity in unrelated individuals of European Ancestries using PLINK.

| | | | In people without obesity (BMI<30) N=256,373 | | In people with obesity (BMI>=30) N=81,163 | | Heterogeneity |
|---|---|---|---|---|---|---|---|
| SNP | pos | Nearest Gene(s) | BETA (SE) | P | BETA (SE) | P | P* |
| rs2787120 | 1:33306297 | *S100PBP* | -0.008 (0.002) | 4.49E-06 | -0.011 (0.004) | 0.002 | 0.502 |
| rs12140153 | 1:62579891 | *PATJ* | -0.015 (0.002) | 1.29E-10 | -0.024 (0.005) | 1.13E-06 | 0.095 |
| rs17131124 | 1:91127548 | *ZNF326, BARHL2* | 0.012 (0.002) | 1.46E-06 | 0.014 (0.005) | 0.003 | 0.710 |
| rs57746981 | 1:201885234 | *LMOD1* | -0.005 (0.001) | 1.93E-04 | -0.005 (0.003) | 0.073 | 1.000 |
| rs825127 | 1:223506788 | *SUSD4* | -0.007 (0.001) | 5.64E-08 | -0.004 (0.003) | 0.150 | 0.343 |
| rs4665972 | 2:27598097 | *SNX17* | 0.007 (0.001) | 1.69E-07 | 0.008 (0.003) | 0.003 | 0.752 |
| rs7598712 | 2:46660452 | *TMEM247* | -0.006 (0.001) | 1.57E-05 | -0.008 (0.003) | 0.003 | 0.527 |
| rs6741951 | 2:58959112 | *LOC644456, LOC730134* | -0.006 (0.001) | 7.16E-05 | -0.008 (0.003) | 0.004 | 0.527 |
| rs11123962 | 2:104157011 | *LOC728815, LOC644265* | 0.006 (0.001) | 2.37E-05 | 0.014 (0.003) | 2.79E-07 | 0.011 |
| rs9712275 | 2:198907143 | *PLCL1* | -0.006 (0.001) | 1.20E-05 | -0.005 (0.003) | 0.068 | 0.752 |
| rs7607363 | 2:213402705 | *ERBB4* | 0.004 (0.001) | 1.15E-03 | 0.006 (0.003) | 0.030 | 0.527 |
| rs13010456 | 2:236792801 | *AGAP1* | -0.009 (0.001) | 5.94E-11 | -0.009 (0.003) | 0.001 | 1.000 |
| rs13097760 | 3:82823561 | *GBE1, CYP51P1* | 0.006 (0.001) | 5.32E-06 | 0.005 (0.003) | 0.101 | 0.752 |
| rs9875075† | 3:84563848 | *CYP51P1, LOC100131101* | 0.005 (0.001) | 3.02E-04 | 0.008 (0.003) | 0.006 | 0.343 |
| rs960986 | 3:85519305 | *LOC440970, CADM2* | -0.005 (0.001) | 3.97E-04 | -0.011 (0.003) | 6.28E-05 | 0.058 |
| rs843372 | 3:183996213 | *ECE2* | 0.007 (0.002) | 6.08E-06 | 0.009 (0.003) | 0.007 | 0.579 |
| rs11942333 | 4:46389486 | *GABRA2* | 0.005 (0.001) | 1.57E-04 | 0.007 (0.003) | 0.018 | 0.527 |
| rs13135092 | 4:103198082 | *SLC39A8* | 0.009 (0.002) | 4.13E-04 | 0.008 (0.005) | 0.078 | 0.853 |
| rs6897863 | 5:92512481 | *CCT7P2, LOC391811* | -0.006 (0.001) | 1.92E-05 | -0.011 (0.003) | 2.59E-05 | 0.114 |
| rs12153518 | 5:138501494 | *SIL1* | 0.007 (0.001) | 9.10E-07 | 0.008 (0.003) | 0.003 | 0.752 |
| rs6923811 | 6:27289776 | *POM121L2, FKSG83* | -0.007 (0.001) | 4.03E-06 | -0.01 (0.003) | 0.0006181 | 0.343 |
| rs55960940 | 6:38153146 | *BTBD9* | -0.006 (0.002) | 6.31E-04 | -0.011 (0.004) | 0.003 | 0.264 |
| rs3122170 | 6:55058998 | *HCRTR2* | 0.009 (0.002) | 8.78E-09 | 0.008 (0.003) | 0.009 | 0.782 |
| rs62519825 | 8:65479707 | *LOC100129963* | 0.009 (0.002) | 1.62E-05 | 0.009 (0.004) | 0.039 | 1.000 |
| rs285793 | 8:106087862 | *LOC644103, LOC100128132* | 0.007 (0.001) | 9.20E-07 | 0.008 (0.003) | 0.003 | 0.752 |
| rs7837226 | 8:131235895 | *ASAP1* | -0.006 (0.001) | 1.92E-06 | -0.005 (0.003) | 0.043 | 0.752 |
| rs55818482 | 9:81744922 | *KRT18P24, CHCHD9* | 0.011 (0.002) | 1.89E-11 | 0.009 (0.003) | 0.007 | 0.579 |
| rs1566362 | 9:128163132 | *GAPVD1, MAPKAP1* | -0.008 (0.001) | 6.36E-09 | -0.004 (0.003) | 0.185 | 0.206 |
| rs7476897 | 10:92416402 | *LOC119358, HTR7* | -0.007 (0.001) | 9.03E-07 | -0.008 (0.003) | 0.003 | 0.752 |
| rs4765939 | 12:2582397 | *CACNA1C* | 0.005 (0.001) | 7.95E-04 | 0.004 (0.003) | 0.100 | 0.752 |
| rs1846644 | 12:117938380 | *KSR2* | 0.01 (0.001) | 5.61E-15 | 0.011 (0.003) | 9.01E-05 | 0.752 |
| rs8015449 | 14:82161860 | *EEF1A1P2, RPL9P6* | -0.005 (0.001) | 7.75E-05 | -0.005 (0.003) | 0.053 | 1.000 |
| rs17356118 | 15:83237899 | *CPEB1* | 0.007 (0.002) | 3.00E-05 | 0.013 (0.003) | 6.86E-05 | 0.096 |
| rs886114 | 16:23865986 | *PRKCB* | 0.005 (0.001) | 7.12E-04 | 0.004 (0.003) | 0.151 | 0.752 |
| rs11649804† | 17:17696755 | *RAI1* | -0.007 (0.001) | 3.05E-06 | -0.003 (0.003) | 0.243 | 0.206 |
| rs62055936† | 17:43848761 | *LOC644191, MGC57346* | -0.009 (0.002) | 4.97E-09 | -0.007 (0.003) | 0.022 | 0.579 |
| rs2048522 | 18:44800515 | *FUSSEL18, TPMTP1* | -0.005 (0.001) | 8.32E-05 | -0.007 (0.003) | 0.013 | 0.527 |
| rs641498 | 6:124911565 | *NKAIN2* | -0.008 (0.001) | 2.60E-08 | -0.005 (0.003) | 0.061 | 0.343 |
| rs12253139 | 10:128899947 | *DOCK1* | 0.008 (0.002) | 3.27E-05 | 0.006 (0.004) | 0.119 | 0.655 |
| rs7982022 | 13:54049003 | *LOC100133285, RP11-365K22.1* | 0.006 (0.001) | 2.98E-05 | 0.006 (0.003) | 0.029 | 1.000 |
| rs2472297 | 15:75027880 | *CYP1A1, CYP1A2* | -0.006 (0.001) | 2.91E-05 | -0.007 (0.003) | 0.018 | 0.752 |
| rs7162082 | 15:83896608 | *HDGFRP3, BNC1* | -0.006 (0.002) | 5.78E-04 | -0.007 (0.003) | 0.041 | 0.782 |

*Heterogeneity between stratified samples was tested using METAL software [Willer et al., 2010].
†For SNPs reported in BOLT analysis (Supplementary Table 3) but not available in PLINK analysis because of filtering criteria differences, proxy SNPs with complete LD (r2=1) in 1000 Genome EUR reference panel was chosen instead.
Red cells: P<0.05/42
Green cells: Heterogeneity P<0.05; No significant heterogeneity after adjusting for multiple comparisons.

**Supplementary Table 6.** Stratified analysis of significant loci on autosomes by sleep duration in unrelated individuals of European Ancestries using PLINK.

| | | | In long sleepers (>8hr) N=25,272 | | In short sleepers (<7hr) N=78,393 | | Heterogeneity |
|---|---|---|---|---|---|---|---|
| SNP | pos | Nearest Gene(s) | BETA (SE) | P | BETA (SE) | P | P* |
| rs2787120 | 1:33306297 | *S100PBP* | -0.009 (0.007) | 0.175 | -0.004 (0.004) | 0.301 | 0.535 |
| rs12140153 | 1:62579891 | *PATJ* | -0.009 (0.009) | 0.346 | -0.018 (0.005) | 1.58E-04 | 0.382 |
| rs17131124 | 1:91127548 | *ZNF326, BARHL2* | 0.019 (0.009) | 0.046 | 0.012 (0.005) | 0.011 | 0.497 |
| rs57746981 | 1:201885234 | *LMOD1* | -0.018 (0.005) | 8.76E-04 | -0.007 (0.003) | 0.018 | 0.059 |
| rs825127 | 1:223506788 | *SUSD4* | -0.013 (0.005) | 0.010 | -0.007 (0.003) | 0.009 | 0.304 |
| rs4665972 | 2:27598097 | *SNX17* | 0.013 (0.005) | 0.015 | 0.001 (0.003) | 0.646 | 0.040 |
| rs7598712 | 2:46660452 | *TMEM247* | -0.014 (0.005) | 0.008 | -0.004 (0.003) | 0.113 | 0.086 |
| rs6741951 | 2:58959112 | *LOC644456, LOC730134* | -0.002 (0.006) | 0.684 | -0.004 (0.003) | 0.189 | 0.766 |
| rs11123962 | 2:104157011 | *LOC728815, LOC644265* | 0.014 (0.005) | 0.008 | 0.009 (0.003) | 0.001 | 0.391 |
| rs9712275 | 2:198907143 | *PLCL1* | -0.007 (0.005) | 0.148 | -0.007 (0.003) | 0.008 | 1.000 |
| rs7607363 | 2:213402705 | *ERBB4* | 0.007 (0.005) | 0.162 | 0.003 (0.003) | 0.240 | 0.493 |
| rs13010456 | 2:236792801 | *AGAP1* | -0.011 (0.005) | 0.041 | -0.01 (0.003) | 3.49E-04 | 0.864 |
| rs13097760 | 3:82823561 | *GBE1, CYP51P1* | 0.01 (0.005) | 0.067 | 0.004 (0.003) | 0.207 | 0.304 |
| rs9875075† | 3:84563848 | *CYP51P1, LOC100131101* | 0.01 (0.006) | 0.083 | 0.004 (0.003) | 0.201 | 0.371 |
| rs960986 | 3:85519305 | *LOC440970, CADM2* | -0.013 (0.005) | 0.013 | -0.006 (0.003) | 0.048 | 0.230 |
| rs843372 | 3:183996213 | *ECE2* | 0.009 (0.006) | 0.135 | 0.003 (0.003) | 0.286 | 0.371 |
| rs11942333 | 4:46389486 | *GABRA2* | 0.014 (0.006) | 0.013 | 0.006 (0.003) | 0.027 | 0.233 |
| rs13135092 | 4:103198082 | *SLC39A8* | 0.014 (0.009) | 0.127 | 0.011 (0.005) | 0.017 | 0.771 |
| rs6897863 | 5:92512481 | *CCT7P2, LOC391811* | -0.005 (0.005) | 0.297 | -0.011 (0.003) | 9.33E-05 | 0.304 |
| rs12153518 | 5:138501494 | *SIL1* | 0.008 (0.005) | 0.130 | 0.006 (0.003) | 0.023 | 0.732 |
| rs6923811 | 6:27289776 | *POM121L2, FKSG83* | 0.001 (0.005) | 0.835 | -0.01 (0.003) | 5.20E-04 | 0.059 |
| rs55960940 | 6:38153146 | *BTBD9* | -0.016 (0.007) | 0.017 | -0.012 (0.004) | 0.001 | 0.620 |
| rs3122170 | 6:55058998 | *HCRTR2* | 0.017 (0.006) | 0.005 | 0.008 (0.003) | 0.012 | 0.180 |
| rs62519825 | 8:65479707 | *LOC100129963* | 0.009 (0.008) | 0.245 | 0.014 (0.004) | 7.36E-04 | 0.576 |
| rs285793 | 8:106087862 | *LOC644103, LOC100128132* | 0.001 (0.005) | 0.876 | 0.009 (0.003) | 9.28E-04 | 0.170 |
| rs7837226 | 8:131235895 | *ASAP1* | -0.007 (0.005) | 0.206 | -0.011 (0.003) | 3.12E-05 | 0.493 |
| rs55818482 | 9:81744922 | *KRT18P24, CHCHD9* | 0.01 (0.006) | 0.132 | 0.012 (0.003) | 1.80E-04 | 0.766 |
| rs1566362 | 9:128163132 | *GAPVD1, MAPKAP1* | -0.009 (0.005) | 0.084 | -0.008 (0.003) | 0.003 | 0.864 |
| rs7476897 | 10:92416402 | *LOC119358, HTR7* | -0.007 (0.005) | 0.184 | -0.009 (0.003) | 0.001 | 0.732 |
| rs4765939 | 12:2582397 | *CACNA1C* | 0.007 (0.005) | 0.183 | 0.004 (0.003) | 0.103 | 0.607 |
| rs1846644 | 12:117938380 | *KSR2* | 0.009 (0.005) | 0.097 | 0.016 (0.003) | 2.45E-09 | 0.230 |
| rs8015449 | 14:82161860 | *EEF1A1P2, RPL9P6* | -0.007 (0.005) | 0.165 | -0.006 (0.003) | 0.028 | 0.864 |
| rs17356118 | 15:83237899 | *CPEB1* | 0.006 (0.006) | 0.359 | 0.013 (0.003) | 5.81E-05 | 0.297 |
| rs886114 | 16:23865986 | *PRKCB* | 0.003 (0.005) | 0.550 | 0.004 (0.003) | 0.179 | 0.864 |
| rs11649804† | 17:17696755 | *RAI1* | -0.007 (0.006) | 0.203 | -0.009 (0.003) | 0.003 | 0.766 |
| rs62055936† | 17:43848761 | *LOC644191, MGC57346* | -0.006 (0.006) | 0.356 | -0.01 (0.003) | 0.002 | 0.551 |
| rs2048522 | 18:44800515 | *FUSSEL18, TPMTP1* | -0.004 (0.005) | 0.475 | -0.004 (0.003) | 0.202 | 1.000 |
| rs641498 | 6:124911565 | *NKAIN2* | -0.011 (0.005) | 0.044 | -0.008 (0.003) | 0.003 | 0.607 |
| rs12253139 | 10:128899947 | *DOCK1* | -0.003 (0.008) | 0.730 | 0.013 (0.004) | 0.002 | 0.074 |
| rs7982022 | 13:54049003 | *LOC100133285, RP11-365K22.1* | 0.01 (0.005) | 0.062 | 0.004 (0.003) | 0.118 | 0.304 |
| rs2472297 | 15:75027880 | *CYP1A1, CYP1A2* | -0.002 (0.006) | 0.777 | -0.005 (0.003) | 0.112 | 0.655 |
| rs7162082 | 15:83896608 | *HDGFRP3, BNC1* | -0.019 (0.006) | 0.003 | -0.005 (0.003) | 0.146 | 0.037 |

*Heterogeneity between stratified samples was tested using METAL software [Willer et al., 2010].
†For SNPs reported in BOLT analysis (Supplementary Table 3) but not available in PLINK analysis because of filtering criteria differences, proxy SNPs with complete LD (r2=1) in 1000 Genome EUR reference panel was chosen instead.
Red cells: P<0.05/42
Green cells: Heterogeneity P<0.05; No significant heterogeneity after adjusting for multiple comparisons.

**Supplementary Table 7.** Associations of significant loci excluding shift workers and people with chronic or psychiatric problems among unrelated individuals of European Ancestries using PLINK.

| SNP | Chr:position (NCBI Build 37) | Nearest Gene(s) | Baseline (N=337539) | | Additional exclusions (N=255426)* | | Inter-action P† |
|---|---|---|---|---|---|---|---|
| | | | BETA (SE) | P | BETA (SE) | P | |
| rs2787120 | 1:33306297 | *S100PBP* | -0.009 (0.002) | $9.70\times10^{-9}$ | -0.011 (0.002) | $9.30\times10^{-10}$ | 0.595 |
| rs12140153 | 1:62579891 | *PATJ* | -0.018 (0.002) | $5.45\times10^{-17}$ | -0.015 (0.002) | $5.35\times10^{-11}$ | 0.036 |
| rs17131124 | 1:91127548 | *ZNF326, BARHL2* | 0.012 (0.002) | $9.66\times10^{-9}$ | 0.011 (0.002) | $7.23\times10^{-6}$ | 0.043 |
| rs57746981 | 1:201885234 | *LMOD1* | -0.006 (0.001) | $8.19\times10^{-6}$ | -0.005 (0.001) | $1.17\times10^{-4}$ | 0.933 |
| rs825127 | 1:223506788 | *SUSD4* | -0.006 (0.001) | $6.69\times10^{-8}$ | -0.005 (0.001) | $1.43\times10^{-4}$ | 0.212 |
| rs4665972 | 2:27598097 | *SNX17* | 0.007 (0.001) | $8.19\times10^{-9}$ | 0.006 (0.001) | $3.55\times10^{-6}$ | 0.141 |
| rs7598712 | 2:46660452 | *TMEM247* | -0.006 (0.001) | $1.44\times10^{-7}$ | -0.007 (0.001) | $1.26\times10^{-6}$ | 0.295 |
| rs6741951 | 2:58959112 | *LOC644456, LOC730134* | -0.006 (0.001) | $9.90\times10^{-6}$ | -0.007 (0.001) | $3.83\times10^{-6}$ | 0.240 |
| rs11123962 | 2:104157011 | *LOC728815, LOC644265* | 0.008 (0.001) | $5.95\times10^{-11}$ | 0.007 (0.001) | $5.78\times10^{-7}$ | 0.309 |
| rs9712275 | 2:198907143 | *PLCL1* | -0.006 (0.001) | $7.18\times10^{-7}$ | -0.006 (0.001) | $1.84\times10^{-6}$ | 0.207 |
| rs7607363 | 2:213402705 | *ERBB4* | 0.004 (0.001) | $2.86\times10^{-4}$ | 0.004 (0.001) | $3.81\times10^{-3}$ | 0.894 |
| rs13010456 | 2:236792801 | *AGAP1* | -0.009 (0.001) | $9.86\times10^{-14}$ | -0.009 (0.001) | $5.59\times10^{-11}$ | 0.708 |
| rs13097760 | 3:82823561 | *GBE1, CYP51P1* | 0.006 (0.001) | $3.52\times10^{-6}$ | 0.006 (0.001) | $5.27\times10^{-5}$ | 0.969 |
| rs9875075‡ | 3:84563848 | *CYP51P1, LOC100131101* | 0.006 (0.001) | $9.77\times10^{-6}$ | 0.006 (0.001) | $7.54\times10^{-5}$ | 0.686 |
| rs960986 | 3:85519305 | *LOC440970, CADM2* | -0.007 (0.001) | $4.93\times10^{-8}$ | -0.007 (0.001) | $1.03\times10^{-6}$ | 0.391 |
| rs843372 | 3:183996213 | *ECE2* | 0.008 (0.001) | $3.44\times10^{-8}$ | 0.007 (0.002) | $1.11\times10^{-5}$ | 0.009 |
| rs11942333 | 4:46389486 | *GABRA2* | 0.006 (0.001) | $5.51\times10^{-6}$ | 0.005 (0.001) | $1.17\times10^{-4}$ | 0.511 |
| rs13135092 | 4:103198082 | *SLC39A8* | 0.010 (0.002) | $7.96\times10^{-6}$ | 0.011 (0.002) | $1.47\times10^{-5}$ | 0.298 |
| rs6897863 | 5:92512481 | *CCT7P2, LOC391811* | -0.007 (0.001) | $2.61\times10^{-9}$ | -0.006 (0.001) | $6.30\times10^{-6}$ | 0.476 |
| rs12153518 | 5:138501494 | *SIL1* | 0.007 (0.001) | $1.66\times10^{-8}$ | 0.007 (0.001) | $3.23\times10^{-7}$ | 0.858 |
| rs6923811 | 6:27289776 | *POM121L2, FKSG83* | -0.007 (0.001) | $2.93\times10^{-8}$ | -0.007 (0.001) | $1.06\times10^{-6}$ | 0.628 |
| rs55960940 | 6:38153146 | *BTBD9* | -0.007 (0.002) | $4.82\times10^{-6}$ | -0.007 (0.002) | $2.28\times10^{-5}$ | 0.769 |
| rs3122170 | 6:55058998 | *HCRTR2* | 0.009 (0.001) | $2.55\times10^{-10}$ | 0.010 (0.002) | $1.14\times10^{-9}$ | 0.216 |
| rs62519825 | 8:65479707 | *LOC100129963* | 0.009 (0.002) | $1.04\times10^{-6}$ | 0.009 (0.002) | $2.67\times10^{-5}$ | 0.947 |
| rs285793 | 8:106087862 | *LOC644103, LOC100128132* | 0.007 (0.001) | $5.31\times10^{-9}$ | 0.007 (0.001) | $4.49\times10^{-8}$ | 0.558 |
| rs7837226 | 8:131235895 | *ASAP1* | -0.006 (0.001) | $2.35\times10^{-7}$ | -0.008 (0.001) | $5.20\times10^{-9}$ | 0.003 |
| rs55818482 | 9:81744922 | *KRT18P24, CHCHD9* | 0.010 (0.001) | $3.12\times10^{-12}$ | 0.010 (0.002) | $2.05\times10^{-9}$ | 0.811 |
| rs1566362 | 9:128163132 | *GAPVD1, MAPKAP1* | -0.007 (0.001) | $2.59\times10^{-8}$ | -0.005 (0.001) | $6.99\times10^{-5}$ | 0.085 |
| rs7476897 | 10:92416402 | *LOC119358, HTR7* | -0.007 (0.001) | $5.56\times10^{-9}$ | -0.008 (0.001) | $7.69\times10^{-8}$ | 0.747 |
| rs4765939 | 12:2582397 | *CACNA1C* | 0.005 (0.001) | $1.62\times10^{-4}$ | 0.004 (0.001) | $4.95\times10^{-3}$ | 0.834 |
| rs1846644 | 12:117938380 | *KSR2* | 0.011 (0.001) | $2.52\times10^{-18}$ | 0.011 (0.001) | $6.73\times10^{-16}$ | 0.269 |
| rs8015449 | 14:82161860 | *EEF1A1P2, RPL9P6* | -0.005 (0.001) | $6.78\times10^{-6}$ | -0.005 (0.001) | $6.40\times10^{-4}$ | 0.082 |
| rs17356118 | 15:83237899 | *CPEB1* | 0.008 (0.001) | $1.94\times10^{-8}$ | 0.007 (0.002) | $5.25\times10^{-6}$ | 0.655 |
| rs886114 | 16:23865986 | *PRKCB* | 0.005 (0.001) | $2.06\times10^{-4}$ | 0.005 (0.001) | $1.76\times10^{-4}$ | 0.839 |
| rs11649804‡ | 17:17696755 | *RAI1* | -0.006 (0.001) | $2.82\times10^{-6}$ | -0.005 (0.001) | $1.07\times10^{-3}$ | 0.072 |
| rs62055936‡ | 17:43848761 | *LOC644191, MGC57346* | -0.009 (0.001) | $1.83\times10^{-9}$ | -0.008 (0.002) | $2.14\times10^{-6}$ | 0.850 |
| rs2048522 | 18:44800515 | *FUSSEL18, TPMTP1* | -0.006 (0.001) | $1.07\times10^{-6}$ | -0.005 (0.001) | $7.56\times10^{-6}$ | 0.537 |
| rs641498 | 6:124911565 | *NKAIN2* | -0.007 (0.001) | $4.13\times10^{-8}$ | -0.007 (0.001) | $6.04\times10^{-7}$ | 0.587 |
| rs12253139 | 10:128899947 | *DOCK1* | 0.008 (0.002) | $2.26\times10^{-5}$ | 0.009 (0.002) | $1.36\times10^{-5}$ | 0.701 |
| rs7982022 | 13:54049003 | *LOC100133285, RP11-365K22.1* | 0.006 (0.001) | $4.25\times10^{-6}$ | 0.005 (0.001) | $9.17\times10^{-5}$ | 0.291 |
| rs2472297 | 15:75027880 | *CYP1A1, CYP1A2* | -0.006 (0.001) | $5.77\times10^{-6}$ | -0.007 (0.001) | $3.34\times10^{-6}$ | 0.026 |
| rs7162082 | 15:83896608 | *HDGFRP3, BNC1* | -0.006 (0.001) | $8.99\times10^{-5}$ | -0.004 (0.002) | $9.07\times10^{-3}$ | 0.145 |
| rs13242253 | 7:84667057 | *SEMA3D* | 0.004 (0.001) | $6.66\times10^{-4}$ | 0.008 (0.001) | $2.40\times10^{-8}$ | $1.56\times10^{-6}$ |

*Additional exclusions include shift workers, people with chronic and psychiatric illness. †SNP and health status (chronic/psychiatric illness) interaction. Red cells: Interaction P<0.05. Significant interaction observed at SEMA3D after adjusting for multiple comparisons. ‡For SNPs reported in BOLT analysis (Supplementary Table 3) but not available in PLINK analysis because of filtering criteria differences, proxy SNPs with complete LD (r2=1) in 1000 Genome EUR reference panel was chosen instead.

**Supplementary Table 8.** Sex-stratified analyses identified additional genome-wide significant loci.

| SNP | Chr:position NCBI Build 37 | Nearest Gene(s) | Alleles (E/A) | EAF | INFO | Sex | β | P | SNP×Sex Interaction P |
|---|---|---|---|---|---|---|---|---|---|
| rs12659640 | 5:64167202 | CWC27 | G/A | 0.6 | 1 | Females | -0.008 (0.001) | **$1.50 \times 10^{-8}$** | 0.033 |
| | | | | | | Males | -0.003 (0.002) | 0.10 | |
| rs9528022 | 13:60226817 | DIAPH3 | T/G | 0.71 | 0.99 | Females | 0.008 (0.002) | **$2.7 \times 10^{-8}$** | 0.228 |
| | | | | | | Males | 0.003 (0.002) | 0.09 | |

**Supplementary Table 9.** Descriptive characteristics of samples from HUNT.

| | Self-reported Daytime Sleepiness | | |
|---|---|---|---|
| | Never/seldom (N=8613) | Sometimes (N=17586) | Several times a week (N=3707) |
| Male, N (%) | 4348 (50.5%) | 8077 (45.9%) | 1459 (39.4%) |
| Age (years), mean (SD) | 55.41 (13.94) | 50.9 (14.92) | 45.06 (14.76) |
| BMI (kg/m2), mean (SD) | 26.87 (3.95) | 26.91 (4.22) | 26.91 (4.22) |

**Supplementary Table 10.** Descriptive characteristics of samples from HEALTH2000.

| | Excessive Daytime Sleepiness | |
|---|---|---|
| | ESS<10 (N=4831) | ESS>10 (N=446) |
| Male, N (%) | 2028 (89.9) | 229 (10.1) |
| Age (years), mean (SD) | 51.5 (14.3) | 56.2 (14.4) |
| BMI (kg/m2), mean (SD) | 24.2 (7.5) | 23.3 (8.1) |

**Supplementary Table 11.** Associations of 42 loci with self-reported daytime sleepiness in HUNT and Health 2000.

| SNP | Nearest Gene(s) | Alleles E/A) | Sign* | HUNT (N=29,906) | | | Health 2000 ESS (N=4,546) | | | Fisher's P-value |
|---|---|---|---|---|---|---|---|---|---|---|
| | | | | β | P | Power | β | P | Power | |
| rs2787120 | *S100PBP* | A/G | + | -0.002 (0.007) | 0.752 | 0.220 | 0.009 (0.085) | 0.915 | 0.051 | 0.946 |
| rs12140153 | *PATJ* | G/T | + | 0.008 (0.008) | 0.300 | 0.520 | -0.065 (0.124) | 0.602 | 0.053 | 0.490 |
| rs17131124 | *ZNF326, BARHL2* | C/G | - | -0.003 (0.009) | 0.699 | 0.237 | -0.088 (0.107) | 0.411 | 0.051 | 0.646 |
| rs57746981 | *LMOD1* | C/T | + | 0.001 (0.005) | 0.915 | 0.263 | -0.020 (0.068) | 0.768 | 0.051 | 0.950 |
| rs825127 | *SUSD4* | T/G | + | 0.006 (0.005) | 0.237 | 0.219 | 0.134 (0.067) | 0.046 | 0.051 | 0.060 |
| rs4665972 | *SNX17* | T/C | + | -0.002 (0.006) | 0.669 | 0.270 | 0.097 (0.069) | 0.159 | 0.051 | 0.345 |
| rs7598712 | *TMEM247* | G/T | + | 0.005 (0.005) | 0.318 | 0.217 | -0.032 (0.066) | 0.630 | 0.051 | 0.522 |
| rs6741951 | *LOC644456, LOC730134* | G/A | + | 0.013 (0.005) | 0.021 | 0.240 | -0.141 (0.076) | 0.063 | 0.051 | 0.010 |
| rs11123962 | *LOC728815, LOC644265* | T/G | - | -0.005 (0.005) | 0.305 | 0.348 | -0.019 (0.067) | 0.783 | 0.052 | 0.581 |
| rs9712275 | *PLCL1* | C/T | - | 0.002 (0.005) | 0.771 | 0.219 | 0.080 (0.069) | 0.247 | 0.051 | 0.506 |
| rs7607363 | *ERBB4* | A/G | - | -0.008 (0.005) | 0.141 | 0.217 | -0.110 (0.067) | 0.103 | 0.051 | 0.076 |
| rs13010456 | *AGAP1* | A/G | + | -0.006 (0.005) | 0.265 | 0.339 | 0.030 (0.071) | 0.669 | 0.052 | 0.484 |
| rs13097760 | *GBE1, CYP51P1* | A/C | - | -0.003 (0.005) | 0.544 | 0.206 | -0.052 (0.070) | 0.456 | 0.051 | 0.594 |
| rs34478464 | *CYP51P1, LOC100131101* | C/T | - | -0.005 (0.007) | 0.440 | 0.285 | -0.023 (0.081) | 0.777 | 0.051 | 0.709 |
| rs960986 | *LOC440970, CADM2* | C/T | + | 0.007 (0.005) | 0.200 | 0.263 | 0.106 (0.067) | 0.116 | 0.051 | 0.110 |
| rs843372 | *ECE2* | C/T | + | 0.010 (0.006) | 0.115 | 0.264 | -0.027 (0.074) | 0.715 | 0.051 | 0.288 |
| rs11942333 | *GABRA2* | G/A | - | 0.004 (0.005) | 0.485 | 0.197 | 0.026 (0.078) | 0.741 | 0.051 | 0.727 |
| rs13135092 | *SLC39A8* | A/G | - | 0.018 (0.0011) | 0.095 | 0.188 | 0.010 (0.258) | 0.968 | 0.051 | 0.311 |
| rs6897863 | *CCT7P2, LOC391811* | A/C | + | 0.001 (0.005) | 0.891 | 0.215 | -0.031 (0.066) | 0.645 | 0.051 | 0.893 |
| rs12153518 | *SIL1* | A/C | + | -0.001 (0.005) | 0.921 | 0.280 | 0.025 (0.071) | 0.725 | 0.051 | 0.938 |
| rs6923811 | *POM121L2, FKSG83* | T/C | + | 0.011 (0.006) | 0.067 | 0.251 | -0.051 (0.088) | 0.557 | 0.051 | 0.160 |
| rs55960940 | *BTBD9* | T/C | + | 0.008 (0.007) | 0.246 | 0.228 | 0.023 (0.084) | 0.784 | 0.051 | 0.511 |
| rs3122170 | *HCRTR2* | C/A | + | 0.001 (0.007) | 0.840 | 0.382 | 0.054 (0.088) | 0.543 | 0.052 | 0.814 |
| rs62519825 | *LOC100129963* | T/C | - | 0.014 (0.008) | 0.093 | 0.199 | -0.084 (0.095) | 0.380 | 0.051 | 0.153 |
| rs285793 | *LOC644103, LOC100128132* | G/A | + | 0.005 (0.005) | 0.296 | 0.280 | 0.156 (0.067) | 0.020 | 0.051 | 0.037 |
| rs7837226 | *ASAP1* | A/G | - | -0.002 (0.005) | 0.739 | 0.219 | -0.007 (0.066) | 0.912 | 0.051 | 0.940 |
| rs55818482 | *KRT18P24, CHCHD9* | T/C | - | -0.001 (0.006) | 0.935 | 0.362 | 0.058 (0.090) | 0.516 | 0.052 | 0.834 |
| rs1566362 | *GAPVD1, MAPKAP1* | T/C | + | -0.001 (0.005) | 0.876 | 0.208 | 0.023 (0.068) | 0.732 | 0.051 | 0.926 |
| rs7476897 | *LOC119358, HTR7* | G/A | + | 0.005 (0.005) | 0.329 | 0.251 | 0.112 (0.079) | 0.152 | 0.051 | 0.200 |
| rs4765939 | *CACNA1C* | G/C | - | 0.007 (0.005) | 0.175 | 0.215 | 0.082 (0.073) | 0.260 | 0.051 | 0.186 |
| rs1846644 | *KSR2* | T/C | - | -0.013 (0.005) | 0.012 | 0.569 | -0.123 (0.067) | 0.069 | 0.053 | 0.007 |
| rs8015449 | *EEF1A1P2, RPL9P6* | A/G | + | 0.006 (0.005) | 0.234 | 0.218 | -0.033 (0.067) | 0.620 | 0.051 | 0.426 |
| rs17356118 | *CPEB1* | A/G | - | -0.010 (0.006) | 0.095 | 0.264 | -0.148 (0.090) | 0.101 | 0.051 | 0.054 |
| rs886114 | *PRKCB* | C/T | + | 0.002 (0.005) | 0.757 | 0.206 | 0.068 (0.073) | 0.349 | 0.051 | 0.616 |
| rs11078398 | *RAI1* | G/A | + | -0.002 (0.006) | 0.711 | 0.282 | NA | NA | 0.051 | 0.282 |
| rs62066119 | *LOC644191, MGC57346* | C/T | + | NA | NA | NA | NA | NA | 0.051 | NA |
| rs2048522 | *FUSSEL18, TPMTP1* | A/T | + | 0.006 (0.005) | 0.234 | 0.216 | -0.025 (0.066) | 0.706 | 0.051 | 0.463 |
| rs641498 | *NKAIN2* | A/G | - | 0.004 (0.005) | 0.482 | 0.211 | -0.042 (0.071) | 0.552 | 0.051 | 0.619 |
| rs12253139 | *DOCK1* | T/C | - | 0.007 (0.007) | 0.365 | 0.211 | -0.054 (0.107) | 0.612 | 0.051 | 0.559 |
| rs7982022 | *LOC100133285, RP11-365K22.1* | G/A | - | -0.002 (0.005) | 0.738 | 0.217 | -0.048 (0.067) | 0.477 | 0.051 | 0.720 |
| rs2472297 | *CYP1A1, CYP1A2* | C/T | + | 0.000 (0.006) | 0.995 | 0.180 | 0.162 (0.083) | 0.050 | 0.051 | 0.199 |
| rs7162082 | *HDGFRP3, BNC1* | C/T | + | 0.006 (0.007) | 0.390 | 0.197 | -0.019 (0.098) | 0.845 | 0.051 | 0.695 |
| Genetic risk score of 42 EDS loci | | | + | 0.361 (0.118) | 0.002 | | 4.045 (1.631) | 0.013 | | $3\times10^{-4}$ |

* Sign of association beta of the effect allele in the UKB discovery analysis.

**Supplementary Table 12.** Descriptive characteristics of samples from FINRISK.

| | Self-reported exhaustion | | |
|---|---|---|---|
| | Never (N=7092) | Sometimes (N=12321) | Several times a week (N=2422) |
| Male, N (%) | 3939 (38.3) | 5444 (53.0) | 890 (8.7) |
| Age (years), mean (SD) | 49.8 (13.7) | 47.4 (12.9) | 47.7 (11.8) |
| BMI (kg/m2), mean (SD) | 26.6 (4.3) | 26.7 (4.7) | 27.1 (5.2) |

**Supplementary Table 13.** Descriptive characteristics of samples from Finnish Twin Cohort Study.

| | Self-reported daytime fatigue | | |
|---|---|---|---|
| | Low (N=3179) | Intermediate (N=1461) | High (N=1126) |
| Male, N (%) | 1589 (50.0) | 618 (42.3) | 418 (37.1) |
| Age (years), mean (SD) | 46.9 (7.6) | 45.9 (7.6) | 49.0 (7.7) |
| BMI (kg/m2), mean (SD) | 24.9 (3.6) | 24.9 (4.1) | 25.8 (5.9) |

**Supplementary Table 14.** Associations of 42 loci with self-reported tiredness in FINRISK and Finnish Twin Study.

| SNP | Nearest Gene(s) | Alleles E/A) | Sign* | FINRISK (N=20,344) β | P | Power | Finnish Twin (N=5,766) β | P | Power | Fisher's P-value |
|---|---|---|---|---|---|---|---|---|---|---|
| rs2787120 | *S100PBP* | A/G | + | -0.001 (0.008) | 0.884 | 0.166 | 0.015 (0.019) | 0.435 | 0.069 | 0.946 |
| rs12140153 | *PATJ* | G/T | + | -0.010 (0.011) | 0.361 | 0.385 | NA | NA | 0.107 | 0.490 |
| rs17131124 | *ZNF326, BARHL2* | C/G | - | -0.014 (0.010) | 0.151 | 0.177 | -0.031 (0.024) | 0.197 | 0.071 | 0.646 |
| rs57746981 | *LMOD1* | C/T | + | NA | NA | 0.195 | 0.034 (0.016) | 0.031 | 0.074 | 0.950 |
| rs825127 | *SUSD4* | T/G | + | NA | NA | 0.165 | -0.008 (0.016) | 0.605 | 0.069 | 0.060 |
| rs4665972 | *SNX17* | T/C | + | 0.001 (0.006) | 0.852 | 0.200 | 0.014 (0.016) | 0.389 | 0.075 | 0.345 |
| rs7598712 | *TMEM247* | G/T | + | -0.012 (0.006) | 0.045 | 0.164 | 0.007 (0.015) | 0.639 | 0.069 | 0.522 |
| rs6741951 | *LOC644456, LOC730134* | G/A | + | 0.005 (0.007) | 0.503 | 0.180 | 0.004 (0.018) | 0.819 | 0.072 | 0.010 |
| rs11123962 | *LOC728815, LOC644265* | T/G | - | 0.002 (0.006) | 0.803 | 0.255 | 0.006 (0.016) | 0.705 | 0.084 | 0.581 |
| rs9712275 | *PLCL1* | C/T | - | 0.001 (0.006) | 0.871 | 0.165 | -0.005 (0.016) | 0.767 | 0.069 | 0.506 |
| rs7607363 | *ERBB4* | A/G | - | -0.008 (0.006) | 0.178 | 0.164 | -0.007 (0.016) | 0.667 | 0.069 | 0.076 |
| rs13010456 | *AGAP1* | A/G | + | -0.002 (0.006) | 0.760 | 0.249 | -0.027 (0.016) | 0.101 | 0.083 | 0.484 |
| rs13097760 | *GBE1, CYP51P1* | A/C | - | -0.001 (0.007) | 0.924 | 0.156 | 0.020 (0.016) | 0.234 | 0.068 | 0.594 |
| rs34478464 | *CYP51P1, LOC100131101* | C/T | - | -0.009 (0.008) | 0.251 | 0.211 | -0.003 (0.019) | 0.884 | 0.077 | 0.709 |
| rs960986 | *LOC440970, CADM2* | C/T | + | -0.008 (0.006) | 0.176 | 0.195 | 0.014 (0.016) | 0.385 | 0.074 | 0.110 |
| rs843372 | *ECE2* | C/T | + | -0.002 (0.007) | 0.712 | 0.196 | -0.008 (0.017) | 0.621 | 0.074 | 0.288 |
| rs11942333 | *GABRA2* | G/A | - | -0.002 (0.007) | 0.740 | 0.150 | 0.031 (0.017) | 0.075 | 0.067 | 0.727 |
| rs13135092 | *SLC39A8* | A/G | - | -0.027 (0.025) | 0.288 | 0.144 | 0.069 (0.06) | 0.250 | 0.066 | 0.311 |
| rs6897863 | *CCT7P2, LOC391811* | A/C | + | -0.004 (0.006) | 0.530 | 0.162 | -0.007 (0.015) | 0.640 | 0.069 | 0.893 |
| rs12153518 | *SIL1* | A/C | + | 0.002 (0.006) | 0.743 | 0.208 | 0.026 (0.016) | 0.093 | 0.076 | 0.938 |
| rs6923811 | *POM121L2, FKSG83* | T/C | + | 0.003 (0.008) | 0.758 | 0.187 | -0.005 (0.02) | 0.819 | 0.073 | 0.160 |
| rs55960940 | *BTBD9* | T/C | + | -0.006 (0.007) | 0.389 | 0.171 | -0.010 (0.019) | 0.596 | 0.070 | 0.511 |
| rs3122170 | *HCRTR2* | C/A | + | 0.0005 (0.008) | 0.977 | 0.280 | -0.053 (0.021) | 0.012 | 0.089 | 0.814 |
| rs62519825 | *LOC100129963* | T/C | - | 0.007 (0.009) | 0.390 | 0.151 | 0.009 (0.022) | 0.684 | 0.067 | 0.153 |
| rs285793 | *LOC644103, LOC100128132* | G/A | + | 0.016 (0.006) | 0.010 | 0.207 | 0.015 (0.016) | 0.334 | 0.076 | 0.037 |
| rs7837226 | *ASAP1* | A/G | - | -0.016 (0.006) | 0.007 | 0.165 | 0.005 (0.015) | 0.732 | 0.069 | 0.940 |
| rs55818482 | *KRT18P24, CHCHD9* | T/C | - | 0.000 (0.008) | 0.955 | 0.265 | 0.028 (0.02) | 0.159 | 0.086 | 0.834 |
| rs1566362 | *GAPVD1, MAPKAP1* | T/C | + | 0.001 (0.006) | 0.822 | 0.157 | 0.00002 (0.016) | 0.999 | 0.068 | 0.926 |
| rs7476897 | *LOC119358, HTR7* | G/A | + | -0.010 (0.007) | 0.200 | 0.187 | 0.003 (0.019) | 0.893 | 0.073 | 0.200 |
| rs4765939 | *CACNA1C* | G/C | - | 0.003 (0.007) | 0.632 | 0.162 | -0.009 (0.017) | 0.571 | 0.069 | 0.186 |
| rs1846644 | *KSR2* | T/C | - | -0.002 (0.006) | 0.735 | 0.424 | 0.012 (0.016) | 0.442 | 0.114 | 0.007 |
| rs8015449 | *EEF1A1P2, RPL9P6* | A/G | + | 0.004 (0.006) | 0.558 | 0.164 | 0.004 (0.015) | 0.812 | 0.069 | 0.426 |
| rs17356118 | *CPEB1* | A/G | - | -0.012 (0.008) | 0.162 | 0.196 | 0.003 (0.021) | 0.894 | 0.074 | 0.054 |
| rs886114 | *PRKCB* | C/T | + | -0.002 (0.007) | 0.720 | 0.156 | -0.015 (0.017) | 0.398 | 0.068 | 0.616 |
| rs11078398 | *RAI1* | G/A | + | -0.002 (0.007) | 0.743 | 0.209 | -0.011 (0.018) | 0.542 | 0.077 | 0.282 |
| rs62066119 | *LOC644191, MGC57346* | C/T | + | -0.002 (0.012) | 0.847 | 0.205 | NA | NA | 0.076 | NA |
| rs2048522 | *FUSSEL18, TPMTP1* | A/T | + | 0.005 (0.006) | 0.448 | 0.163 | -0.021 (0.016) | 0.182 | 0.069 | 0.463 |
| rs641498 | *NKAIN2* | A/G | - | -0.008 (0.007) | 0.219 | 0.160 | -0.005 (0.016) | 0.765 | 0.068 | 0.619 |
| rs12253139 | *DOCK1* | T/C | - | 0.017 (0.01) | 0.096 | 0.159 | 0.014 (0.025) | 0.577 | 0.068 | 0.559 |
| rs7982022 | *LOC100133285, RP11-365K22.1* | G/A | - | 0.009 (0.006) | 0.146 | 0.164 | -0.001 (0.016) | 0.925 | 0.069 | 0.720 |
| rs2472297 | *CYP1A1, CYP1A2* | C/T | + | 0.007 (0.007) | 0.310 | 0.138 | 0.011 (0.018) | 0.561 | 0.065 | 0.199 |
| rs7162082 | *HDGFRP3, BNC1* | C/T | + | 0.011 (0.009) | 0.221 | 0.150 | 0.003 (0.023) | 0.879 | 0.067 | 0.695 |
| Genetic risk score of 42 EDS loci | | | + | 0.099 (0.148) | 0.504 | | -0.3 (0.382) | 0.443 | | 0.551 |

* Sign of association beta of the effect allele in the UKB discovery analysis.

**Supplementary Table 15.** Significant Gene-based association analysis using PASCAL after adjusting for multiple comparisons (enrichment $P<2.29\times10^{-6}$).

| chromosome | start | end | strand | Gene ID | Gene Symbol | SNP in Gene | P-value |
|---|---|---|---|---|---|---|---|
| chr1 | 201798287 | 201853422 | + | 55705 | IPO9 | 212 | 7.37E-12 |
| chr1 | 201857796 | 201861715 | + | 149345 | SHISA4 | 145 | 4.93E-11 |
| chr1 | 201777738 | 201777830 | + | 1E+08 | MIR1231 | 124 | 5.03E-11 |
| chr3 | 184043483 | 184043559 | + | 692107 | SNORD66 | 133 | 5.07E-11 |
| chr17 | 43922255 | 43924438 | + | 162540 | SPPL2C | 176 | 1.02E-10 |
| chr17 | 43920721 | 43972879 | - | 1E+08 | MAPT-AS1 | 193 | 1.11E-10 |
| chr17 | 44076615 | 44077060 | + | 246744 | STH | 20 | 1.26E-10 |
| chr3 | 184017021 | 184026840 | + | 5708 | PSMD2 | 134 | 1.31E-10 |
| chr3 | 184032282 | 184053146 | + | 1981 | EIF4G1 | 160 | 1.32E-10 |
| chr17 | 43697709 | 43913194 | + | 1394 | CRHR1 | 230 | 1.34E-10 |
| chr17 | 43971747 | 44105699 | + | 4137 | MAPT | 48 | 1.90E-10 |
| chr1 | 62208148 | 62644347 | + | 10207 | INADL | 1204 | 2.23E-10 |
| chr17 | 43973148 | 43976164 | + | 1E+08 | MAPT-IT1 | 25 | 2.90E-10 |
| chr6 | 55039070 | 55147418 | + | 3062 | HCRTR2 | 443 | 3.09E-10 |
| chr17 | 44107281 | 44302740 | - | 284058 | KANSL1 | 50 | 3.63E-10 |
| chr17 | 43578683 | 43597889 | - | 55073 | LRRC37A4P | 19 | 3.89E-10 |
| chr17 | 43677490 | 43679748 | - | 644172 | LOC644172 | 7 | 8.26E-10 |
| chr17 | 43513265 | 43568146 | - | 9842 | PLEKHM1 | 38 | 1.47E-09 |
| chr17 | 43471267 | 43511112 | - | 201176 | ARHGAP27 | 37 | 1.78E-09 |
| chr12 | 117890816 | 118406028 | - | 283455 | KSR2 | 1589 | 1.79E-09 |
| chr17 | 43716340 | 43723595 | + | 147081 | CRHR1-IT1 | 11 | 1.88E-09 |
| chr1 | 201865583 | 201915716 | - | 25802 | LMOD1 | 295 | 5.07E-09 |
| chr15 | 82944749 | 83182973 | - | 727751 | LOC727751 | 21 | 5.12E-09 |
| chr15 | 82763612 | 83182973 | - | 80154 | LOC80154 | 22 | 5.49E-09 |
| chr17 | 44668034 | 44834828 | + | 4905 | NSF | 92 | 1.05E-08 |
| chr6 | 55192266 | 55267291 | + | 389400 | GFRAL | 497 | 1.07E-08 |
| chr9 | 128024110 | 128127290 | + | 26130 | GAPVD1 | 224 | 1.93E-08 |
| chr17 | 44839871 | 44896126 | - | 7473 | WNT3 | 150 | 1.94E-08 |
| chr2 | 198591602 | 198650938 | - | 66037 | BOLL | 141 | 4.46E-08 |
| chr1 | 201617449 | 201796102 | + | 89796 | NAV1 | 459 | 4.47E-08 |
| chr15 | 82821160 | 83209208 | - | 1.01E+08 | RPS17L | 67 | 5.02E-08 |
| chr2 | 198570027 | 198573114 | + | 92935 | MARS2 | 111 | 5.28E-08 |
| chr1 | 33327868 | 33338082 | - | 252995 | FNDC5 | 101 | 6.84E-08 |
| chr17 | 17679999 | 17682843 | - | 140771 | SMCR5 | 94 | 7.34E-08 |
| chr1 | 33352097 | 33360247 | + | 3208 | HPCA | 111 | 9.15E-08 |
| chr9 | 128199672 | 128469513 | - | 79109 | MAPKAP1 | 343 | 9.52E-08 |
| chr17 | 44270938 | 44274089 | + | 644246 | KANSL1-AS1 | 6 | 9.70E-08 |
| chr2 | 198669425 | 199014608 | + | 5334 | PLCL1 | 581 | 9.86E-08 |
| chr1 | 33240839 | 33283633 | - | 8565 | YARS | 150 | 1.04E-07 |
| chr1 | 33207511 | 33240571 | + | 57648 | KIAA1522 | 128 | 1.16E-07 |
| chr2 | 236402732 | 237040444 | + | 116987 | AGAP1 | 1407 | 1.20E-07 |
| chr1 | 33283042 | 33324480 | + | 64766 | S100PBP | 140 | 1.21E-07 |
| chr2 | 27714749 | 27718126 | - | 64838 | FNDC4 | 102 | 1.23E-07 |
| chr2 | 27719705 | 27746550 | + | 2646 | GCKR | 133 | 1.51E-07 |
| chr17 | 44363861 | 44657088 | - | 51326 | ARL17A | 18 | 1.53E-07 |
| chr3 | 184053716 | 184064063 | + | 131408 | FAM131A | 142 | 1.73E-07 |
| chr10 | 128594022 | 129250780 | + | 1793 | DOCK1 | 1914 | 1.80E-07 |
| chr8 | 131094983 | 131097014 | - | 1.01E+08 | LOC100507117 | 187 | 2.06E-07 |
| chr1 | 33360195 | 33366953 | - | 113452 | TMEM54 | 110 | 2.14E-07 |
| chr2 | 27667239 | 27712571 | - | 26160 | IFT172 | 136 | 2.47E-07 |
| chr17 | 44372496 | 44415160 | + | 9884 | LRRC37A | 13 | 3.13E-07 |
| chr2 | 58747887 | 59290901 | + | 400955 | FLJ30838 | 1027 | 3.20E-07 |
| chr8 | 130851838 | 131028897 | - | 51571 | FAM49B | 512 | 3.43E-07 |
| chr8 | 131064350 | 131455906 | - | 50807 | ASAP1 | 969 | 3.97E-07 |
| chr9 | 127997126 | 128003666 | - | 3309 | HSPA5 | 129 | 4.96E-07 |
| chr15 | 82555151 | 82577267 | + | 283726 | FAM154B | 135 | 5.28E-07 |
| chr8 | 131307600 | 131308779 | - | 29065 | ASAP1-IT1 | 175 | 6.08E-07 |

| | | | | | | | |
|---|---|---|---|---|---|---|---|
| chr3 | 85008132 | 86123579 | + | 253559 | CADM2 | 2002 | 6.38E-07 |
| chr10 | 128933689 | 128994422 | - | 642938 | FAM196A | 291 | 6.85E-07 |
| chr1 | 223394160 | 223537544 | - | 55061 | SUSD4 | 287 | 7.09E-07 |
| chr2 | 46706703 | 46711564 | + | 388946 | TMEM247 | 181 | 7.66E-07 |
| chr15 | 82422560 | 82555104 | - | 79631 | EFTUD1 | 391 | 8.62E-07 |
| chr1 | 223566714 | 223568812 | + | 164127 | C1orf65 | 129 | 9.00E-07 |
| chr2 | 212240441 | 213403352 | - | 2066 | ERBB4 | 3177 | 9.01E-07 |
| chr2 | 198435526 | 198540584 | - | 130132 | RFTN2 | 202 | 9.01E-07 |
| chr15 | 82585620 | 82924242 | + | 390660 | LOC390660 | 76 | 9.50E-07 |
| chr3 | 85849136 | 85877200 | - | 1.01E+08 | CADM2-AS2 | 174 | 9.53E-07 |
| chr2 | 27548715 | 27579901 | - | 2976 | GTF3C2 | 75 | 9.64E-07 |
| chr8 | 65492794 | 65496191 | + | 27319 | BHLHE22 | 129 | 9.88E-07 |
| chr2 | 27665232 | 27669348 | + | 200634 | KRTCAP3 | 92 | 1.02E-06 |
| chr2 | 27558408 | 27560670 | + | 1.01E+08 | LOC100505624 | 55 | 1.02E-06 |
| chr1 | 62660473 | 62678001 | + | 54596 | L1TD1 | 369 | 1.04E-06 |
| chr15 | 83394649 | 83408532 | - | 283693 | LOC283693 | 155 | 1.06E-06 |
| chr18 | 25530929 | 25757445 | - | 1000 | CDH2 | 475 | 1.08E-06 |
| chr2 | 27650656 | 27665124 | + | 29959 | NRBP1 | 97 | 1.15E-06 |
| chr16 | 23847299 | 24231932 | + | 5579 | PRKCB | 1074 | 1.16E-06 |
| chr3 | 183967444 | 184010819 | + | 9718 | ECE2 | 161 | 1.23E-06 |
| chr2 | 27587218 | 27593324 | - | 8890 | EIF2B4 | 60 | 1.24E-06 |
| chr1 | 33402049 | 33430286 | - | 127544 | RNF19B | 134 | 1.28E-06 |
| chr18 | 44738459 | 44775554 | - | 652991 | SKOR2 | 227 | 1.38E-06 |
| chr2 | 27600097 | 27603611 | - | 130557 | ZNF513 | 70 | 1.38E-06 |
| chr2 | 27604065 | 27632550 | - | 5496 | PPM1G | 90 | 1.42E-06 |
| chr8 | 65486865 | 65494445 | - | 401463 | LOC401463 | 140 | 1.43E-06 |
| chr2 | 27593362 | 27600400 | + | 9784 | SNX17 | 67 | 1.44E-06 |
| chr15 | 83211950 | 83316728 | - | 64506 | CPEB1 | 201 | 1.45E-06 |
| chr2 | 27615489 | 27616443 | - | 2498 | FTH1P3 | 76 | 1.46E-06 |
| chr15 | 83379222 | 83382778 | - | 338963 | LOC338963 | 135 | 1.55E-06 |
| chr5 | 138282409 | 138534065 | - | 64374 | SIL1 | 343 | 1.56E-06 |
| chr9 | 127962820 | 127996438 | + | 10244 | RABEPK | 176 | 1.63E-06 |
| chr6 | 54711568 | 54809897 | + | 222584 | FAM83B | 504 | 1.68E-06 |
| chr11 | 134022336 | 134094426 | - | 23310 | NCAPD3 | 243 | 1.69E-06 |
| chr7 | 132937822 | 133750513 | + | 60412 | EXOC4 | 1261 | 2.03E-06 |
| chr11 | 133938819 | 134021652 | + | 83700 | JAM3 | 337 | 2.08E-06 |
| chr11 | 133902166 | 133911236 | + | 1E+08 | LOC100128239 | 233 | 2.27E-06 |

**Supplementary Table 16.** Tissue enrichment analysis of gene expression in GTEx RNA-seq data using MAGMA.

| Tissue | BETA | SE | P |
|---|---|---|---|
| **Brain_Cerebellar_Hemisphere** | **0.048** | **0.008** | **$1.74{\times}10^{-9}$** |
| **Brain_Cerebellum** | **0.046** | **0.008** | **$1.44{\times}10^{-8}$** |
| **Brain_Frontal_Cortex_BA9** | **0.045** | **0.009** | **$3.79{\times}10^{-7}$** |
| **Brain_Anterior_cingulate_cortex_BA24** | **0.047** | **0.010** | **$6.30{\times}10^{-7}$** |
| **Brain_Cortex** | **0.043** | **0.009** | **$2.62{\times}10^{-6}$** |
| **Brain_Nucleus_accumbens_basal_ganglia** | **0.041** | **0.010** | **$1.84{\times}10^{-5}$** |
| **Brain_Hypothalamus** | **0.044** | **0.011** | **$2.18{\times}10^{-5}$** |
| **Brain_Amygdala** | **0.039** | **0.011** | **$1.47{\times}10^{-4}$** |
| **Brain_Hippocampus** | **0.038** | **0.011** | **$2.37{\times}10^{-4}$** |
| **Brain_Caudate_basal_ganglia** | **0.034** | **0.011** | **$5.80{\times}10^{-4}$** |
| **Brain_Putamen_basal_ganglia** | **0.033** | **0.011** | **$8.62{\times}10^{-4}$** |
| Brain_Substantia_nigra | 0.030 | 0.012 | 0.005 |
| Pituitary | 0.026 | 0.012 | 0.016 |
| Testis | 0.014 | 0.007 | 0.021 |
| Brain_Spinal_cord_cervical_c.1 | 0.023 | 0.012 | 0.024 |
| Cells_EBV.transformed_lymphocytes | -0.001 | 0.007 | 0.567 |
| Muscle_Skeletal | -0.002 | 0.009 | 0.594 |
| Adrenal_Gland | -0.006 | 0.013 | 0.683 |
| Cells_Transformed_fibroblasts | -0.006 | 0.010 | 0.739 |
| Colon_Sigmoid | -0.013 | 0.018 | 0.765 |
| Ovary | -0.014 | 0.013 | 0.858 |
| Whole_Blood | -0.008 | 0.007 | 0.859 |
| Esophagus_Muscularis | -0.019 | 0.018 | 0.861 |
| Esophagus_Gastroesophageal_Junction | -0.021 | 0.019 | 0.873 |
| Heart_Left_Ventricle | -0.014 | 0.012 | 0.887 |
| Artery_Tibial | -0.019 | 0.014 | 0.904 |
| Pancreas | -0.016 | 0.012 | 0.910 |
| Liver | -0.011 | 0.008 | 0.918 |
| Nerve_Tibial | -0.022 | 0.015 | 0.921 |
| Heart_Atrial_Appendage | -0.020 | 0.013 | 0.942 |
| Esophagus_Mucosa | -0.016 | 0.010 | 0.943 |
| Uterus | -0.029 | 0.016 | 0.962 |
| Stomach | -0.032 | 0.017 | 0.970 |
| Kidney_Cortex | -0.024 | 0.013 | 0.972 |
| Small_Intestine_Terminal_Ileum | -0.022 | 0.011 | 0.973 |
| Skin_Sun_Exposed_Lower_leg | -0.022 | 0.011 | 0.980 |
| Skin_Not_Sun_Exposed_Suprapubic | -0.022 | 0.011 | 0.981 |
| Cervix_Ectocervix | -0.042 | 0.020 | 0.983 |
| Colon_Transverse | -0.033 | 0.015 | 0.986 |
| Artery_Aorta | -0.032 | 0.015 | 0.986 |
| Bladder | -0.040 | 0.018 | 0.989 |
| Thyroid | -0.033 | 0.014 | 0.989 |
| Spleen | -0.023 | 0.010 | 0.990 |
| Artery_Coronary | -0.046 | 0.017 | 0.996 |
| Prostate | -0.048 | 0.018 | 0.997 |
| Vagina | -0.047 | 0.017 | 0.998 |
| Cervix_Endocervix | -0.055 | 0.018 | 0.999 |
| Adipose_Subcutaneous | -0.048 | 0.015 | 0.999 |
| Minor_Salivary_Gland | -0.043 | 0.014 | 0.999 |
| Lung | -0.042 | 0.013 | 0.999 |
| Breast_Mammary_Tissue | -0.061 | 0.019 | 0.999 |
| Fallopian_Tube | -0.067 | 0.018 | 1.000 |
| Adipose_Visceral_Omentum | -0.059 | 0.016 | 1.000 |

MAGMA analysis conditioned on gene size, log gene size, gene density, log gene density, inverse minor allele count, and log inverse minor allele count.

**Supplementary Table 17.** Significant pathways enriched in candidate genes from excessive daytime sleepiness gene loci (PASCAL Bonferroni P<0.05).

| Name | Bonferroni P |
|---|---|
| REACTOME_NEURONAL_SYSTEM | 3.00E-07 |
| REACTOME_TRANSMISSION_ACROSS_CHEMICAL_SYNAPSES | 5.00E-07 |
| REACTOME_NEUROTRANSMITTER_RECEPTOR_BINDING_AND_DOWNSTREAM_TRANSMISSION_IN_THE_POSTSYNAPTIC_CELL | 7.40E-06 |
| REACTOME_CELL_CELL_JUNCTION_ORGANIZATION | 1.05E-04 |
| REACTOME_GABA_RECEPTOR_ACTIVATION | 1.95E-04 |
| REACTOME_ADHERENS_JUNCTIONS_INTERACTIONS | 3.50E-04 |
| REACTOME_CELL_JUNCTION_ORGANIZATION | 6.10E-04 |
| REACTOME_POTASSIUM_CHANNELS | 7.70E-04 |
| REACTOME_TRAFFICKING_OF_GLUR2_CONTAINING_AMPA_RECEPTORS | 1.60E-03 |
| KEGG_ENDOCYTOSIS | 2.04E-03 |
| REACTOME_MEIOSIS | 2.71E-03 |
| KEGG_LONG_TERM_DEPRESSION | 2.93E-03 |
| REACTOME_MEIOTIC_SYNAPSIS | 3.10E-03 |
| REACTOME_GABA_A_RECEPTOR_ACTIVATION | 3.14E-03 |
| REACTOME_DOWNSTREAM_SIGNALING_EVENTS_OF_B_CELL_RECEPTOR_BCR | 3.21E-03 |
| REACTOME_CELL_CELL_COMMUNICATION | 3.30E-03 |
| KEGG_NEUROACTIVE_LIGAND_RECEPTOR_INTERACTION | 3.57E-03 |
| REACTOME_PACKAGING_OF_TELOMERE_ENDS | 3.78E-03 |
| REACTOME_SIGNALING_BY_THE_B_CELL_RECEPTOR_BCR | 4.69E-03 |
| REACTOME_GABA_B_RECEPTOR_ACTIVATION | 4.72E-03 |
| REACTOME_ACTIVATION_OF_NF_KAPPAB_IN_B_CELLS | 4.76E-03 |
| REACTOME_NEUROTRANSMITTER_RELEASE_CYCLE | 5.78E-03 |
| REACTOME_ENDOSOMAL_SORTING_COMPLEX_REQUIRED_FOR_TRANSPORT_ESCRT | 7.26E-03 |
| BIOCARTA_CDMAC_PATHWAY | 8.13E-03 |
| BIOCARTA_NOS1_PATHWAY | 8.58E-03 |
| KEGG_TIGHT_JUNCTION | 8.84E-03 |
| REACTOME_OPIOID_SIGNALLING | 9.68E-03 |
| REACTOME_CDT1_ASSOCIATION_WITH_THE_CDC6_ORC_ORIGIN_COMPLEX | 9.77E-03 |
| REACTOME_MTORC1_MEDIATED_SIGNALLING | 9.83E-03 |
| REACTOME_VOLTAGE_GATED_POTASSIUM_CHANNELS | 9.85E-03 |
| REACTOME_LIGAND_GATED_ION_CHANNEL_TRANSPORT | 0.011 |
| REACTOME_TRAFFICKING_OF_AMPA_RECEPTORS | 0.012 |
| KEGG_MAPK_SIGNALING_PATHWAY | 0.014 |
| REACTOME_G_ALPHA_S_SIGNALLING_EVENTS | 0.014 |
| BIOCARTA_VIP_PATHWAY | 0.015 |
| REACTOME_RNA_POL_I_PROMOTER_OPENING | 0.015 |
| BIOCARTA_BIOPEPTIDES_PATHWAY | 0.015 |
| REACTOME_MYOGENESIS | 0.016 |
| BIOCARTA_MEF2D_PATHWAY | 0.016 |
| BIOCARTA_CALCINEURIN_PATHWAY | 0.016 |
| REACTOME_PI3K_EVENTS_IN_ERBB2_SIGNALING | 0.016 |
| REACTOME_ION_CHANNEL_TRANSPORT | 0.016 |
| BIOCARTA_FMLP_PATHWAY | 0.016 |
| REACTOME_CHROMOSOME_MAINTENANCE | 0.017 |
| REACTOME_SYNTHESIS_OF_DNA | 0.017 |
| BIOCARTA_VEGF_PATHWAY | 0.017 |
| BIOCARTA_GABA_PATHWAY | 0.017 |
| REACTOME_TELOMERE_MAINTENANCE | 0.017 |
| BIOCARTA_TCR_PATHWAY | 0.018 |
| REACTOME_MEMBRANE_TRAFFICKING | 0.019 |
| REACTOME_HEMOSTASIS | 0.019 |
| REACTOME_CDK_MEDIATED_PHOSPHORYLATION_AND_REMOVAL_OF_CDC6 | 0.020 |
| BIOCARTA_EIF4_PATHWAY | 0.020 |

| | |
|---|---|
| BIOCARTA_EIF_PATHWAY | 0.020 |
| BIOCARTA_RANKL_PATHWAY | 0.020 |
| REACTOME_G_ALPHA_Z_SIGNALLING_EVENTS | 0.020 |
| BIOCARTA_NTHI_PATHWAY | 0.020 |
| KEGG_WNT_SIGNALING_PATHWAY | 0.021 |
| REACTOME_MEIOTIC_RECOMBINATION | 0.023 |
| BIOCARTA_EPONFKB_PATHWAY | 0.025 |
| BIOCARTA_RNA_PATHWAY | 0.025 |
| REACTOME_INHIBITION_OF_VOLTAGE_GATED_CA2_CHANNELS_VIA_GBETA_GAMMA_SUBUNITS | 0.026 |
| REACTOME_SCF_BETA_TRCP_MEDIATED_DEGRADATION_OF_EMI1 | 0.027 |
| KEGG_AXON_GUIDANCE | 0.027 |
| REACTOME_S_PHASE | 0.028 |
| REACTOME_INWARDLY_RECTIFYING_K_CHANNELS | 0.029 |
| REACTOME_TRNA_AMINOACYLATION | 0.029 |
| REACTOME_CASPASE_MEDIATED_CLEAVAGE_OF_CYTOSKELETAL_PROTEINS | 0.030 |
| REACTOME_AUTODEGRADATION_OF_THE_E3_UBIQUITIN_LIGASE_COP1 | 0.031 |
| BIOCARTA_P35ALZHEIMERS_PATHWAY | 0.031 |
| REACTOME_ORC1_REMOVAL_FROM_CHROMATIN | 0.031 |
| KEGG_MELANOGENESIS | 0.031 |
| REACTOME_ADENYLATE_CYCLASE_INHIBITORY_PATHWAY | 0.032 |
| REACTOME_REGULATION_OF_ORNITHINE_DECARBOXYLASE_ODC | 0.032 |
| BIOCARTA_P27_PATHWAY | 0.033 |
| KEGG_LONG_TERM_POTENTIATION | 0.033 |
| REACTOME_DEVELOPMENTAL_BIOLOGY | 0.033 |
| KEGG_REGULATION_OF_AUTOPHAGY | 0.034 |
| KEGG_CYTOSOLIC_DNA_SENSING_PATHWAY | 0.036 |
| REACTOME_ASSEMBLY_OF_THE_PRE_REPLICATIVE_COMPLEX | 0.036 |
| REACTOME_VIF_MEDIATED_DEGRADATION_OF_APOBEC3G | 0.037 |
| REACTOME_SCFSKP2_MEDIATED_DEGRADATION_OF_P27_P21 | 0.037 |
| BIOCARTA_LEPTIN_PATHWAY | 0.037 |
| REACTOME_TRIF_MEDIATED_TLR3_SIGNALING | 0.038 |
| KEGG_AMINOACYL_TRNA_BIOSYNTHESIS | 0.038 |
| REACTOME_M_G1_TRANSITION | 0.038 |
| REACTOME_DOPAMINE_NEUROTRANSMITTER_RELEASE_CYCLE | 0.038 |
| BIOCARTA_TALL1_PATHWAY | 0.039 |
| REACTOME_RORA_ACTIVATES_CIRCADIAN_EXPRESSION | 0.041 |
| REACTOME_DESTABILIZATION_OF_MRNA_BY_AUF1_HNRNP_D0 | 0.041 |
| REACTOME_P53_INDEPENDENT_G1_S_DNA_DAMAGE_CHECKPOINT | 0.041 |
| BIOCARTA_AGPCR_PATHWAY | 0.042 |
| BIOCARTA_GPCR_PATHWAY | 0.042 |
| REACTOME_PI3K_EVENTS_IN_ERBB4_SIGNALING | 0.042 |
| BIOCARTA_IL1R_PATHWAY | 0.043 |
| REACTOME_TAK1_ACTIVATES_NFKB_BY_PHOSPHORYLATION_AND_ACTIVATION_OF_IKKS_COMPLEX | 0.043 |
| BIOCARTA_DNAFRAGMENT_PATHWAY | 0.044 |
| REACTOME_GLUTAMATE_NEUROTRANSMITTER_RELEASE_CYCLE | 0.044 |
| REACTOME_APOPTOSIS_INDUCED_DNA_FRAGMENTATION | 0.044 |
| REACTOME_PLC_BETA_MEDIATED_EVENTS | 0.044 |
| BIOCARTA_PGC1A_PATHWAY | 0.047 |
| REACTOME_PKB_MEDIATED_EVENTS | 0.047 |
| BIOCARTA_CELL2CELL_PATHWAY | 0.047 |
| REACTOME_APOPTOTIC_EXECUTION_PHASE | 0.048 |
| REACTOME_TIGHT_JUNCTION_INTERACTIONS | 0.048 |
| REACTOME_P53_DEPENDENT_G1_DNA_DAMAGE_RESPONSE | 0.049 |
| KEGG_PROTEASOME | 0.050 |

**Supplementary Table 18.** Partitioned heritability of self-reported daytime sleepiness across tissue types using LDSC.

| Cell Type Group | Proportion of SNPs | Proportion of heritability | Proportion of heritability SE | Enrichment | Enrichment SE | Enrichment p-value |
|---|---|---|---|---|---|---|
| **Central Nervous System** | **0.149** | **0.345** | **0.042** | **2.316** | **0.282** | **3.28E-06** |
| **Adrenal/Pancreas** | **0.094** | **0.235** | **0.040** | **2.515** | **0.429** | **3.70E-04** |
| Skeletal Muscle | 0.104 | 0.192 | 0.035 | 1.854 | 0.337 | 0.012 |
| Cardiovascular | 0.111 | 0.209 | 0.041 | 1.877 | 0.373 | 0.020 |
| Kidney | 0.043 | 0.099 | 0.027 | 2.326 | 0.634 | 0.036 |
| Connective/Bone | 0.115 | 0.167 | 0.040 | 1.452 | 0.350 | 0.197 |
| Immune | 0.233 | 0.264 | 0.052 | 1.130 | 0.225 | 0.561 |
| Liver | 0.072 | 0.088 | 0.029 | 1.215 | 0.407 | 0.598 |
| Gastrointestinal | 0.168 | 0.152 | 0.048 | 0.908 | 0.287 | 0.748 |

**Supplementary Table 19.** Partitioned heritability of self-reported daytime sleepiness across functional annotation clasases using LDSC.

| Category | Proportion of SNPs | Proportion of heritability | Proportion of heritability SE | Enrichment | Enrichment SE | Enrichment p-value |
|---|---|---|---|---|---|---|
| **29 mammals conserved** | **0.026** | **0.462** | **0.057** | **17.716** | **2.194** | **1.31E-12** |
| **29 mammals conserved, extended** | **0.333** | **0.643** | **0.049** | **1.935** | **0.146** | **2.56E-09** |
| **Intron, extended** | **0.397** | **0.502** | **0.025** | **1.264** | **0.064** | **3.96E-05** |
| **H3K4me1, extended** | **0.609** | **0.797** | **0.049** | **1.308** | **0.081** | **2.62E-04** |
| FetalDHS, extended | 0.285 | 0.489 | 0.067 | 1.717 | 0.235 | 0.003 |
| Coding | 0.015 | 0.093 | 0.026 | 6.320 | 1.773 | 0.004 |
| Weak Enhancer, extended | 0.089 | 0.224 | 0.046 | 2.513 | 0.514 | 0.004 |
| H3K4me3 | 0.133 | 0.266 | 0.048 | 1.997 | 0.358 | 0.005 |
| Repressed, extended | 0.719 | 0.620 | 0.035 | 0.862 | 0.049 | 0.005 |
| Fantom5 Enhancer, extended | 0.154 | 0.281 | 0.056 | 1.823 | 0.361 | 0.025 |
| DHS, extended | 0.499 | 0.690 | 0.087 | 1.384 | 0.173 | 0.025 |
| H3K9ac, extended | 0.231 | 0.345 | 0.053 | 1.498 | 0.228 | 0.032 |
| Enhancer, extended | 0.019 | -0.022 | 0.020 | -1.166 | 1.059 | 0.039 |
| Intron | 0.387 | 0.446 | 0.029 | 1.152 | 0.075 | 0.041 |
| 3' UTR | 0.011 | 0.055 | 0.022 | 4.989 | 2.000 | 0.049 |
| H3K4me1 peaks | 0.171 | 0.335 | 0.085 | 1.956 | 0.496 | 0.055 |
| Super Enhancer, extended | 0.172 | 0.214 | 0.022 | 1.246 | 0.128 | 0.059 |
| CTCF | 0.024 | -0.048 | 0.045 | -2.004 | 1.897 | 0.115 |
| TFBS, extended | 0.343 | 0.452 | 0.070 | 1.315 | 0.204 | 0.127 |
| H3K4me3 peaks | 0.042 | 0.107 | 0.048 | 2.549 | 1.153 | 0.178 |
| Weak Enhancer | 0.021 | 0.074 | 0.039 | 3.491 | 1.849 | 0.179 |
| TSS | 0.018 | 0.056 | 0.029 | 3.055 | 1.565 | 0.191 |
| DGF, extended | 0.542 | 0.635 | 0.080 | 1.172 | 0.149 | 0.246 |
| Fantom5 Enhancer | 0.063 | 0.122 | 0.052 | 1.927 | 0.822 | 0.260 |
| TFBS | 0.132 | 0.215 | 0.081 | 1.620 | 0.614 | 0.314 |
| Promoter, extended | 0.039 | 0.061 | 0.022 | 1.583 | 0.582 | 0.316 |
| Transcribed | 0.345 | 0.420 | 0.082 | 1.216 | 0.237 | 0.358 |
| TSS, extended | 0.035 | 0.061 | 0.028 | 1.743 | 0.810 | 0.360 |
| Super Enhancer | 0.168 | 0.190 | 0.023 | 1.127 | 0.139 | 0.362 |
| 5' UTR | 0.005 | 0.017 | 0.013 | 3.117 | 2.354 | 0.371 |
| **H3K9ac** | 0.126 | 0.168 | 0.056 | 1.329 | 0.445 | 0.458 |
| Promoter | 0.031 | 0.013 | 0.026 | 0.405 | 0.823 | 0.469 |
| 3' UTR extended | 0.027 | 0.042 | 0.021 | 1.570 | 0.786 | 0.472 |
| Coding, extended | 0.065 | 0.083 | 0.028 | 1.292 | 0.430 | 0.497 |
| Transcribed, extended | 0.763 | 0.794 | 0.048 | 1.041 | 0.063 | 0.514 |
| FetalDHS | 0.085 | 0.140 | 0.087 | 1.656 | 1.021 | 0.520 |
| H3K9ac peaks | 0.039 | 0.082 | 0.067 | 2.104 | 1.728 | 0.522 |
| Promoter Flanking | 0.008 | -0.006 | 0.025 | -0.694 | 2.973 | 0.569 |
| H3K27ac | 0.391 | 0.412 | 0.037 | 1.054 | 0.094 | 0.569 |
| **H3K4me1** | 0.427 | 0.461 | 0.072 | 1.080 | 0.169 | 0.635 |
| DHS peaks | 0.112 | 0.068 | 0.096 | 0.610 | 0.860 | 0.650 |
| DHS | 0.168 | 0.124 | 0.101 | 0.742 | 0.604 | 0.669 |
| H3K27ac, extended | 0.423 | 0.439 | 0.041 | 1.040 | 0.096 | 0.680 |
| Promoter Flanking, extended | 0.033 | 0.020 | 0.034 | 0.602 | 1.025 | 0.698 |
| H3K4me3, extended | 0.255 | 0.234 | 0.057 | 0.914 | 0.222 | 0.698 |
| Repressed | 0.461 | 0.436 | 0.094 | 0.944 | 0.204 | 0.785 |
| DGF | 0.138 | 0.148 | 0.095 | 1.075 | 0.693 | 0.914 |
| CTCF, extended | 0.071 | 0.074 | 0.043 | 1.046 | 0.601 | 0.940 |
| 5' UTR extended | 0.028 | 0.027 | 0.021 | 0.978 | 0.739 | 0.977 |
| Enhancer | 0.004 | 0.004 | 0.016 | 1.003 | 3.714 | 0.999 |

**Supplementary Table 20.** Causal links between genetically correlated cardiometabolic, psychiatric, reproductive, and sleep problems and self-reported daytime sleepiness outcome using Mendelian randomization.

| | | | | Inverse Variance Weighted | | |
|---|---|---|---|---|---|---|
| Exposure | Study | Pubmed ID | SNPs | Beta | SE | *p-val* |
| Body mass index (kg/m2) | GIANT | 25673413 | 259 | **0.020** | **0.005** | **0.0001** |
| Type 2 diabetes | DIAGRAM | 24509480 | 58 | **0.005** | **0.002** | **0.012** |
| Coronary heart disease | CARDIoGRAM | 21378990 | 30 | 0.004 | 0.003 | 0.175 |
| Neuroticism | GPC | 25993607 | 3 | 0.008 | 0.024 | 0.733 |
| Bipolar disorder | PGC | 21926972 | 14 | 0.007 | 0.004 | 0.068 |
| Major depressive disorder | PGC | 22472876 | 21 | 0.0003 | 0.001 | 0.794 |
| Schizophrenia | PGC | 25056061 | 95 | 0.013 | 0.011 | 0.236 |
| Menarche (age at onset) | ReproGen | 25231870 | 119 | -0.007 | 0.004 | 0.061 |
| Restless Leg syndrome | EU-RLS GENE, INTERVAL, and 23andMe | 29029846 | 16 | 0.004 | 0.005 | 0.401 |
| Narcolepsy | Immunochip consortium | 23459209 | 5 | 0.002 | 0.006 | 0.784 |
| Insomnia | UK Biobank | | 57 | -0.035 | 0.031 | 0.261 |
| Sleep Duration | UK Biobank | | 78 | -0.0001 | 0.0003 | 0.751 |
| Short Sleep | UK Biobank | | 27 | -0.121 | 0.079 | 0.126 |
| Long Sleep | UK Biobank | | 8 | 0.230 | 0.217 | 0.287 |
| Chronotype | UK Biobank, 23andMe | | 348 | -0.006 | 0.006 | 0.306 |

**Supplementary Table 21.** MR Sensitivity analysis using Radial MR-Egger.

| | Model/parameter | Est. | S.E. | p-value | Heterogeneity statistic | Weak Instrument statistic |
|---|---|---|---|---|---|---|
| **BMI-sleepiness** | IVW | | | | | |
| | $\beta_{IVW}$ | 0.018 | 0.005 | 0.0004 | Cochran's Q = 677.16 ($p = 1.09 \times 10^{-37}$) | Mean F=32.7 |
| | Radial MR-Egger | | | | | |
| | β intercept | -0.142 | 0.297 | 0.633 | | |
| | β slope | 0.025 | 0.015 | 0.103 | Rucker's Q = 676.58 ($p = 8.11 \times 10^{-38}$) | $I^2$=0.89 |
| **T2D-sleepiness** | IVW | | | | | |
| | $\beta_{IVW}$ | 0.005 | 0.002 | 0.014 | Cochran's Q = 88.38 (p = 0.005) | Mean F=29.01 |
| | Radial MR-Egger | | | | | |
| | β intercept | 0.283 | 0.345 | 0.414 | | |
| | β slope | 0.002 | 0.004 | 0.637 | Rucker's Q = 87.32 (p = 0.005) | $I^2$=0.84 |

**Supplementary Table 22**. Mendelian randomization analyses using self-reported daytime sleepiness as exposure.

| | | Sleepiness unadjusted for BMI | | | | Sleepiness adjusted for BMI | | | |
|---|---|---|---|---|---|---|---|---|---|
| Outcome | Pubmed ID | nsnp | b | se | pval | nsnp | b | se | pval |
| Major Depressive Disorder | 29700475 | 34 | 0.09054 | 0.1181 | 0.4435 | 34 | 0.1086 | 0.1249 | 0.3846 |
| Type 2 Diabetes | 24509480 | 28 | 0.1672 | 0.5693 | 0.769 | 32 | 0.01422 | 0.5581 | 0.9797 |
| Neuroticism | 25993607 | 33 | 0.1259 | 0.1515 | 0.4059 | 34 | 0.2431 | 0.1684 | 0.1488 |
| Age at menarche | 25231870 | 28 | -0.1649 | 0.3222 | 0.6088 | 31 | -0.00786 | 0.3208 | 0.9805 |
| Body mass index | 25673413 | 23 | 0.1996 | 0.2712 | 0.4617 | 27 | 0.1975 | 0.234 | 0.3988 |
| Schizophrenia | 25056061 | 33 | 0.7694 | 0.7715 | 0.3186 | 34 | 0.6493 | 0.6531 | 0.3201 |
| Coronary heart disease | 21378990 | 29 | 0.5372 | 0.4857 | 0.268 | 32 | 0.4494 | 0.5033 | 0.3719 |
| Bipolar disorder | 21926972 | 23 | 1.141 | 0.9789 | 0.2437 | 27 | 0.2818 | 0.7653 | 0.7127 |

**Supplementary Note 1.** Medication lists.

**1. Sleep medications:** oxazepam, meprobamate, medazepam, bromazepam, lorazepam, clobazam, chlormezanone, temazepam, nitrazepam, lormetazepam, diazepam, zopiclone, riclofos, methyprylone, prazepam, triazolam, ketazolam, dichloralphenazone, clomethiazole, zaleplon, butobarbital.

**2. Antidepressants:** amitriptyline, citalopram, fluoxetine, sertraline, venlafaxine, dosulepin, paroxetine, mirtazapine, escitalopram, trazodone, prozac, seroxat, cipralex, duloxetine, lofepramine, clomipramine, nortriptyline, imipramine, dothiepin, cipramil, amitriptyline, prothiaden, trimipramine, lustral, reboxetine, zispin, cymbalta, anafranil, doxepin, moclobemide, phenelzine, fluvoxamine, yentreve, triptafen, surmontil, tranylcypromine, allegron, edronax, molipaxin, mianserin, nardil, faverin, nefazodone, amitriptyline+chlordiazepoxide, isocarboxazid, manerix, maoi, sinequan, tranylcypromine+trifluoperazine, ludiomil, norval, tryptizol, and fluphenazine hydrochloride+nortriptyline.

**3. Antipsychotics:** prochlorperazine, olanzapine, quetiapine, risperidone, chlorpromazine, trifluoperazine, amisulpride, sulpiride, seroquel, haloperidol, aripiprazole, stelazine, depixol, flupentixol, clozapine, promazine, risperdal, modecate, fluanxol, flupenthixol, zyprexa, zuclopenthixol, clopixol, largactil, abilify, fluphenazine, haldol, serenace, clozaril, cpz, perphenazine, levomepromazine, pericyazine, dolmatil, fentazin, fluphenazine, benperidol, pimozide, zaponex, denzapine, neulactil, thioridazine, dozic, fluspirilene, panadeine, and sertindole. 4. Anxiolytics: zopiclone, diazepam, temazepam, zolpidem, nitrazepam, lorazepam, hydroxyzine, zimovane, phenergan, promethazine, buspirone, atarax, oxazepam, loprazolam, chlordiazepoxide, lormetazepam, ucerax, stilnoct, diazepam, buspar, alprazolam, librium, xanax, meprate, dalmane, clomethiazole, meprobamate, welldorm, amitriptyline+chlordiazepoxide, flurazepam, heminevrin, medazepam, neulactil, sinequan, almazine, atensine, carisoma, chloractil, chloral, dichloralphenazone, dormonoct, methyprylone, mogadon, rohypnol, tryptizol.

**Supplementary Note 2.** Criteria for identifying participants with psychiatric disorders.

Cases of psychiatric disorder were defined by the following criteria [1]:

1) ICD-10 codes for major depressive disorder (F32, F33), bipolar disorder (F30, F31), schizophrenia (F20-F29), autism (F84.0, F84.3, F84.5), intellectual disability (F70.0, F70.1, F70.9), anxiety disorder (F40 - F43), multiple personality disorder (F44.8), or mood disorder (F30 - F39).

2) Self-reported antidepressant, antipsychotic, or anxiolytic use at nurse-led interview.

3) Self-reported depression, major depressive disorder, bipolar disorder, or schizophrenia at nurse-led interview.

4) "Broad depression": responded yes to the question "Have you ever seen a general practitioner (GP) for nerves, anxiety, tension or depression?" and yes to either, "have you ever had a time when you were feeling depressed or down for at least a whole week," or, "Have you ever had a time when you were uninterested in things or unable to enjoy the things you used to for at least a whole week?" lasting for more than 1 week.

5) Questionnaire-assessed bipolar disorder (Smith algorithm [2]): responded yes to the question, "Have you ever had a period of time lasting at least two days when you were feeling so good, "high", excited or "hyper" that other people thought you were not your normal self or you were so "hyper" that you got into trouble?" or "Have you ever had a period of time lasting at least two days when you were so irritable that you found yourself shouting at people or starting fights or arguments?" for a duration of at least one week and with at least 3 manic/hyper symptoms.

----------------------

1. Howard DM, Adams MJ, Shirali M, et al. Genome-wide association study of depression phenotypes in UK Biobank identifies variants in excitatory synaptic pathways. Nat Commun. 2018; 9 (1): 1470.
2. Smith DJ, Nicholl BI, Cullen B, et al. Prevalence and characteristics of probable major depression and bipolar disorder within UK biobank: cross-sectional study of 172,751 participants. PLoS One. 2013; 8 (11): e75362.
