# Supplementary material for: Genome-wide association analysis of self-reported daytime sleepiness identifies 42 loci that suggest biological subtypes
Source: Nat Commun. 2019 Aug 13;10:3503. doi: 10.1038/s41467-019-11456-7 (PMC6692391; doi:10.1038/s41467-019-11456-7)
Supplement: Supplementary file 8 — Reporting Summary [file 41467_2019_11456_MOESM8_ESM.pdf]

nature research

Corresponding author(s): Richa Saxena

Last updated by author(s): Jun 25, 2019

Reporting Summary

Nature Research wishes to improve the reproducibility of the work that we publish. This form provides structure for consistency and transparency in reporting. For further information on Nature Research policies, see Authors & Referees and the Editorial Policy Checklist.

Statistics

For all statistical analyses, confirm that the following items are present in the figure legend, table legend, main text, or Methods section.

n/a	Confirmed	
☐	☒	The exact sample size (*n*) for each experimental group/condition, given as a discrete number and unit of measurement
☐	☒	A statement on whether measurements were taken from distinct samples or whether the same sample was measured repeatedly
☐	☒	The statistical test(s) used AND whether they are one- or two-sided<br>*Only common tests should be described solely by name; describe more complex techniques in the Methods section.*
☐	☒	A description of all covariates tested
☐	☒	A description of any assumptions or corrections, such as tests of normality and adjustment for multiple comparisons
☐	☒	A full description of the statistical parameters including central tendency (e.g. means) or other basic estimates (e.g. regression coefficient) AND variation (e.g. standard deviation) or associated estimates of uncertainty (e.g. confidence intervals)
☐	☒	For null hypothesis testing, the test statistic (e.g. *F*, *t*, *r*) with confidence intervals, effect sizes, degrees of freedom and *P* value noted<br>*Give P values as exact values whenever suitable.*
☒	☐	For Bayesian analysis, information on the choice of priors and Markov chain Monte Carlo settings
☒	☐	For hierarchical and complex designs, identification of the appropriate level for tests and full reporting of outcomes
☐	☒	Estimates of effect sizes (e.g. Cohen's *d*, Pearson's *r*), indicating how they were calculated

Our web collection on statistics for biologists contains articles on many of the points above.

Software and code

Policy information about availability of computer code

Data collection	All data was collected by the UK Biobank, HUNT study, FINRISK study, Health2000 Survey, and Finnish Twin Cohort. This is a secondary use of data.
Data analysis	The following softwares were used: R 3.12, BOLT-LMM 2.3.2, PLINK 1.9, omconvert, FUSION TWAS, Affymetrix Power Tools 1.16.1, MRbase 1.2.1, LDhub 1.9.0, LDSC 1.0.0, FUMA 1.3.3, MS Office2016, SHAPEIT3, Impute4, GCTA 1.90.0, and Pascal. We used no custom code.

For manuscripts utilizing custom algorithms or software that are central to the research but not yet described in published literature, software must be made available to editors/reviewers. We strongly encourage code deposition in a community repository (e.g. GitHub). See the Nature Research guidelines for submitting code & software for further information.

Data

Policy information about availability of data

All manuscripts must include a data availability statement. This statement should provide the following information, where applicable:

- Accession codes, unique identifiers, or web links for publicly available datasets
- A list of figures that have associated raw data
- A description of any restrictions on data availability

UK Biobank Sleep Traits GWAS summary statistics are available at the Sleep Disorder Knowledge Portal (SDKP) website (http://www.sleepdisordergenetics.org). All other data are contained within the article and its supplementary information or available upon request.

Field-specific reporting

Please select the one below that is the best fit for your research. If you are not sure, read the appropriate sections before making your selection.

☒ Life sciences ☐ Behavioural & social sciences ☐ Ecological, evolutionary & environmental sciences

For a reference copy of the document with all sections, see nature.com/documents/nr-reporting-summary-flat.pdf

Life sciences study design

All studies must disclose on these points even when the disclosure is negative.

Sample size	We used all available subjects in the UK Biobank of European ancestry with complete phenotype and genotype information. European ancestry was determined by cluster analysis of the genetic principal components of ancestry.
Data exclusions	Excluded subjects of non-European ancestry or taking any self-reported sleep medication were excluded.
Replication	We replicated the genetic associations in UK Biobank, HUNT study, FINRISK study, Health2000 Surcey, Finnish Twin Cohort, and UK Biobank accelerometer study.
Randomization	This is not relevant to this study, as there are no groups.
Blinding	This is not relevant to this study, as there are no groups.

Reporting for specific materials, systems and methods

We require information from authors about some types of materials, experimental systems and methods used in many studies. Here, indicate whether each material, system or method listed is relevant to your study. If you are not sure if a list item applies to your research, read the appropriate section before selecting a response.

Materials & experimental systems

n/a	Involved in the study	
☒	☐	Antibodies
☒	☐	Eukaryotic cell lines
☒	☐	Palaeontology
☒	☐	Animals and other organisms
☐	☒	Human research participants
☒	☐	Clinical data

Methods

n/a	Involved in the study	
☒	☐	ChIP-seq
☒	☐	Flow cytometry
☒	☐	MRI-based neuroimaging

Human research participants

Policy information about studies involving human research participants

Population characteristics	See supplementary table 1 for a detailed description of the research participants. Briefly, participants were 45.7% male, 57 years old on average, with 76.8% self-reporting "often", 20.5% self-reporting "sometimes" and 2.7% self-reporting "often" sleepy during the daytime.
Recruitment	Participants were recruited by the UK Biobank by mailers to 9 million people in the UK Medical system. The UK Biobank population is healthier than average with a lower mortality rate.
Ethics oversight	The UK Biobank study was approved by the National Health Service National Research Ethics Service (ref. 11/NW/0382), and all participants provided written informed consent to participate in the UK Biobank study.

Note that full information on the approval of the study protocol must also be provided in the manuscript.
