# Supplementary material for: Genome-wide association analysis of self-reported daytime sleepiness identifies 42 loci that suggest biological subtypes
Source: Nat Commun. 2019 Aug 13;10:3503. doi: 10.1038/s41467-019-11456-7 (PMC6692391; doi:10.1038/s41467-019-11456-7)
Supplement: Supplementary file 9 — Description of Additional Supplementary Files [file 41467_2019_11456_MOESM9_ESM.docx]

**Title:** Supplementary Data 1.
**Description:** Sensitivity analyses of significant loci on autosomes using unrelated individuals of European Ancestries using PLINK (N=337539).

**Title:** Supplementary Data 2.
**Description:** Associations between sleepiness loci and other sleep traits and BMI.

**Title:** Supplementary Data 3.
**Description:** Annotation of genes under association signals for self-reported daytime sleepiness.

**Title:** Supplementary Data 4.
**Description:** Variants annotation for SNPs with a causal probability larger than 0.2.

**Title:** Supplementary Data 5.
**Description:** Pathway and ontology enrichment analysis using EnrichR.

**Title:** Supplementary Data 6.
**Description:** Genetic correlations (LDSC) between self-reported daytime sleepiness and other traits.
